# *Pas de Deux* of an NO Couple: Synchronous Photoswitching from a Double‐Linear to a Double‐Bent Ru(NO)_2_ Core under Nitrosyl Charge Conservation

**DOI:** 10.1002/anie.202210671

**Published:** 2022-09-15

**Authors:** Asma Hasil, Daniel Beck, Daniel Schröder, Sébastien Pillet, Emmanuel Wenger, Theo Woike, Peter Klüfers, Dominik Schaniel

**Affiliations:** ^1^ Université de Lorraine, CNRS, CRM2 54000 Nancy France; ^2^ Department Chemie der Ludwigs-Maximilians-Universität Butenandtstraße 5–13 81377 München Germany

**Keywords:** Coordination Modes, Nitrosyl Ligands, Photo-Induced Linkage Isomerism, Photophysics, Ruthenium

## Abstract

The {Ru(NO)_2_}^10^ dinitrosylruthenium complex [Ru(NO)_2_(PPh_3_)_2_] (**1**) shows photo‐induced linkage isomerism (PLI) of a special kind: the two NO ligands switch, on photo‐excitation, synchronously from the ground state (GS) with two almost linear RuNO functions to a metastable state (MS) which persists up to 230 K and can be populated to ≈50 %. The MS was experimentally characterised by photo‐crystallography, IR spectroscopy and DS‐calorimetry as a double‐bent variant of the double‐linear GS. The experimental results are confirmed by computation which unravels the GS/MS transition as a disrotatory synchronous 50° turn of the two nitrosyl ligands. Although **1** shows the usual redshift of the N−O stretch on bending the MNO unit, there is no increased charge transfer from Ru to NO along the GS‐to‐MS path. In terms of the effective‐oxidation‐state (EOS) method, both isomers of **1** and the transition state are Ru^−II^(NO^+^)_2_ species.

## Graphical abstract

The picture shows the transition state (TS) which connects the double‐linear ground state (GS) with the double‐bent metastable state (MS) of the dinitrosylruthenium compound [Ru(NO)_2_(PPh_3_)_2_] (**1**). The TS's strongly negative vibrational mode (−831 cm^−1^) traces the decay path if the photo‐generated MS is warmed above 230 K. **1** provides the first example of synchronous photo‐induced linkage isomerism (PLI) involving two ligands (see Supporting Information for an animation of the movement).

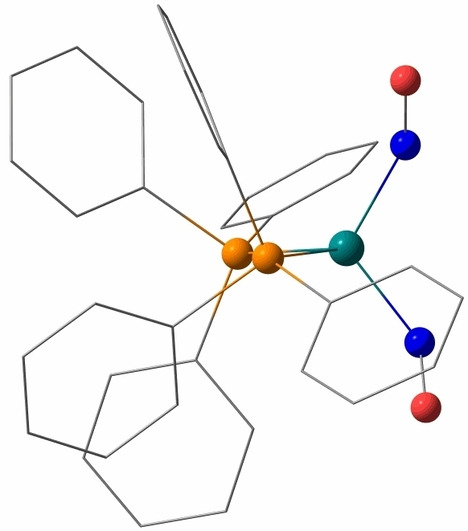

## Introduction

**Scheme 1:**
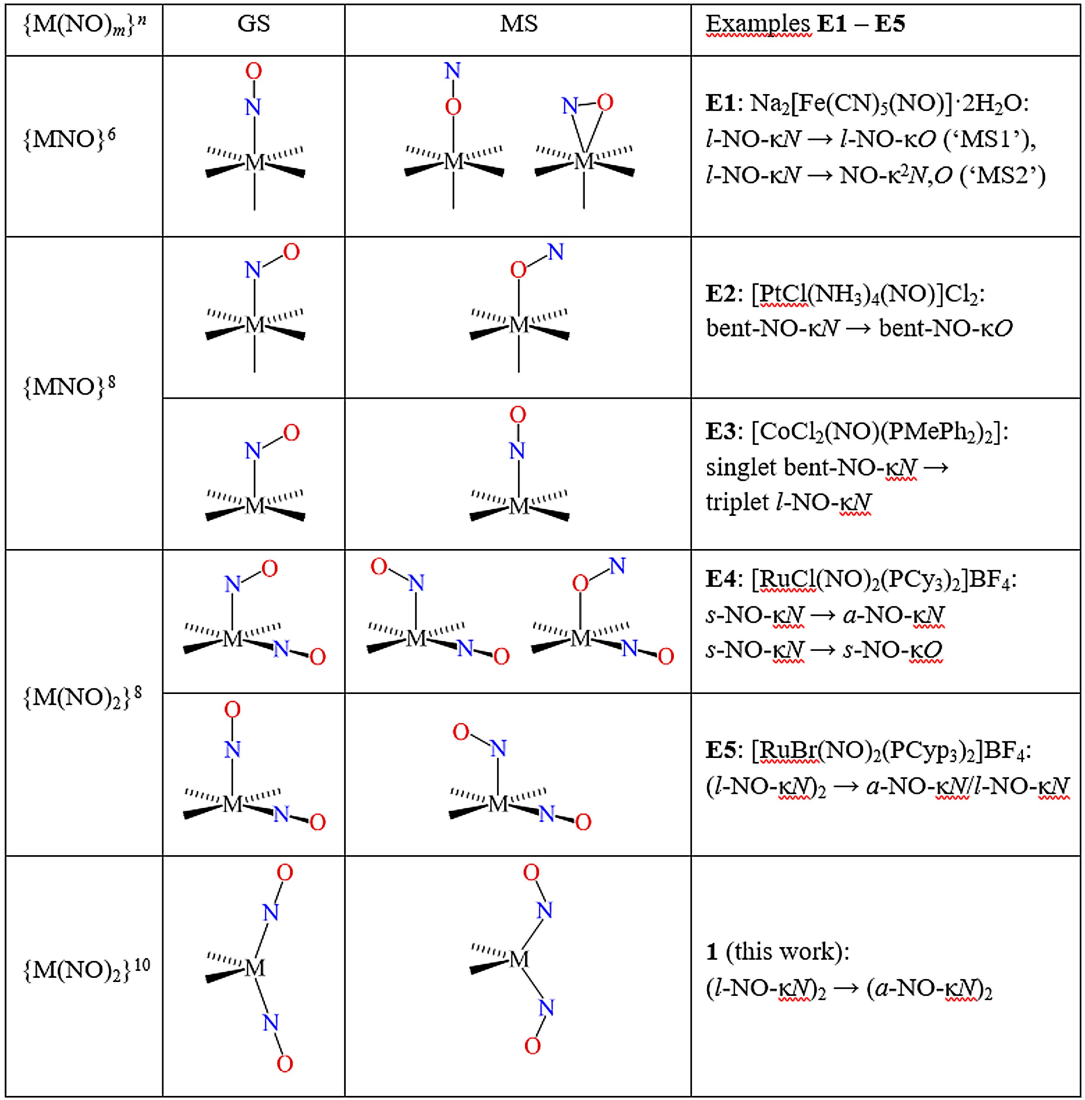
Linkage isomers of mononuclear mono‐ and dinitrosylmetal species.^[5]^

Reversible molecular photoswitches are of great interest in the search for sensitive materials with fast response that can be used for building photonic devices or molecular machines.^[1]^ In this context, photoinduced linkage isomerism (PLI) in transition‐metal nitrosyl compounds is of importance since the reversible photoswitching of the NO ligand is accompanied by significant photochromic and photorefractive changes.^[2]^ In order to better understand the PLI mechanism responsible for these exciting properties, the knowledge of the bonding and activation of NO is essential. Multiple photo‐induced configurations can be generated in mono‐ and dinitrosyl compounds, depending on the electronic configuration of the {MNO}^*n*^ and {M(NO)_2_}^*n*^ systems (the superscript *n* is the number of metal‐d electrons if NO^+^ is assumed to be the ligand: the Enemark–Feltham notation). Scheme 1 shows a compilation of the possible linkage isomers.

Linear MNO bonding is usually the ground state (GS) of {MNO}^*n*^ systems with *n*≤6 (example **E1** in Scheme 1).^[3]^ The prototypical nitroprusside, [Fe(CN)_5_(NO)]^2−^, establishes the linear linkage by the interplay of three interactions: two Fe‐to‐NO π‐backbonds from the low‐spin‐d^6^ metal centre's occupied d(*xz*) and d(*yz*) AOs (*z* running along the Fe−NO axis) to the empty N−O‐π* MOs, and the NO‐to‐Fe donor bond from the N‐atom's lone pair to the iron's empty d(*z* ^2^) AO.^[4]^ It should be noted that the degree of ON→M donation is irrelevant for the linearity of the MNO moiety. Instead, the important point is that on σ‐interaction in an *n*≤6 system there is no repulsion between the N‐centered lone pair and any electrons in the d(*x* ^2^−*y* ^2^)/d(*z* ^2^) set. With its empty d(*z* ^2^) orbital, the low‐spin‐d^6^ ferrous centre is thus a perfect “host” for the triple orbital interaction.

PLI generates variants of this scheme: in the isonitrosyl (MS1), the O‐atom's lone pair interacts with the empty d(*z* ^2^) AO; in the side‐on MS2 isomer, it is the N−O‐π‐bond in the FeNO‐plane, say *xz*, which interacts with the empty d(*z* ^2^) AO. The decisive backbonds form with the same nitrosyl‐MOs in the case of MS1 (though with a smaller overlap and thus less stability), whereas in the MS2 isomer the occupied d(*xz*) cloverleaf at Fe forms a backbond with the in‐plane NO(π*). A second backbond of local π‐symmetry from Fe‐d(*yz*) to the empty NO‐π*(*y*) MO completes the three‐orbital scheme for MS2. On rotation of the NO ligand from the GS via MS2 to MS1, orbital overlap collapses between the three minima which results in sufficient activation barriers along the path as a prerequisite for populating the local MS2 and MS1 minima.

For *n*>6, occupied metal orbitals of the d(*z* ^2^) and d(*x* ^2^−*y* ^2^) type change the scene. Linear GS and MS1 states require an effective avoidance of the surplus metal electrons and the nitrosyl's donor pair, whereas an MS2 is no longer a clear minimum on the energy hypersurface. {MNO}^8^ species such as the long‐known {CoNO}^8^ centres, the configuration of which is mostly derived from the d‐occupation pattern (*xy*)^2^(*xz*)^2^(*yz*)^2^(*z* ^2^)^2^(*x* ^2^−*y* ^2^)^0^, are the best understood electron‐rich systems. The simple nitroprusside bonding scheme is made impossible by the occupied d(*z* ^2^) orbital for two reasons. (1) The d(*z* ^2^) pair competes as a donor pair in a backbond with d(*xz*). (2) As an occupied orbital, it imposes Pauli repulsion with the ligand's lone pairs (which affects the GS and the MS1) or the occupied NO(π) bonds (which affects the MS2). For the GS, the majority of the {MNO}^8^ species choose back‐donation from the d(*z* ^2^) pair (case 1) into one lobe of one of the empty acceptor orbitals. A σ‐bond forms which results in an obtuse MNO angle of 120–140° (without a too pronounced effect on the second backbond). A minority of {MNO}^8^ centres are found, however, with a linear MNO entity by leaving the trans‐to‐NO position empty or weakened. In this bonding mode, metal‐p(*z*) admixture of the next shell to the d(*z* ^2^) orbital polarises the electron pair away from the interfering nitrosyl lone pair. As a result, {MNO}^8^ species establish two M→NO backbonds with either d(*xz*)/d(*yz*) in the linear case, and d(*z* ^2^)/d(*yz*) in the bent class in mostly pentacoordinate or (5+1)‐coordinate species with a sixth ligand at a greater distance.

In summary, we end up, in principle, with four possible linkage isomers for an {MNO}^8^ centre. The linear or the bent κ*N*‐bonding MNO moiety, one of them being the GS, and the N/O‐interchanged κ*O*‐bonding isomers of both. “In principle” points to a frequent limitation. An activation barrier between the linear/bent couples is typically small or non‐existent. As a result, a metastable bent/linear isomer above a linear/bent state, either κ*N* or κ*O*, is not tangible and we are left with only two isomers in total. An example is the {MNO}^8^ species [PtCl(NH_3_)_4_(NO)]^2+^ (example **E2** in Scheme 1).^[6]^ Here, the number of isomers is restricted by the absent metastability of the linear form. On scanning the Pt−N−O angle from the bent GS towards linearity, the energy rises without reaching a local minimum (see Figure S1 in the Supporting Information). As a result, there is a single PL‐isomer, the bent‐κ*O* form which is derived from the bent‐κ*N* GS by the N/O interchange within the nitrosyl.^[6]^ Note that the scene is widened if the multiplicity changes on irradiation (example **E3** in Scheme 1).^[7]^

{Ru(NO)_2_}^8^ dinitrosyls change the energy landscape of the various isomers. {M(NO)_2_}^8^ species can be composed (conceptually, sometimes also in reality) from a square‐planar {MNO}^8^ mononitrosyl and an NO^+^ acceptor ligand. The square planar core (the nitrosyl ligand of which is always linearly bonded) seems to be largely inert in PLI experiments, leaving the second nitrosyl along *z* as the photophysically reactive one. Specifically, the switching of a single nitrosyl on photophysical excitation has been observed in a series of {Ru(NO)_2_}^8^ dinitrosyls of various halogenido‐bis(phosphane)‐ruthenium cores. In a dinitrosyl, one more geometrical parameter has to be accounted for in a bent isomer. The nitrosyl ligand in the bent bonding mode can be inclined either away from (*anti*) or towards (*syn*) the inert nitrosyl. On moving from *anti* to *syn* and back, the active nitrosyl passes a point of linear bonding. In terms of energy, the double‐linear structure is found as a transition or an intermediate state in electron‐poor systems. In electron‐rich species, the double‐linear species is the GS. In this case, photo‐excitation may switch one nitrosyl to the bent mode. Hence, despite the fact that the nitrosyls’ possible bonding modes follow the same linear/bent pattern for {MNO}^8^ and {M(NO)_2_}^8^ species geometrically, energetically we end up with a significant difference. As shown above, activation walls between the bent and the linear isomers are low to non‐existent in the case of an {MNO}^8^ mononitrosyl, but both isomers appear tangible in the {M(NO)_2_}^8^ dinitrosyls for one of the nitrosyl ligands.

Various PLI events have been reported for pentacoordinate {Ru(NO)_2_}^8^ species. (1) [RuCl(NO)_2_(PPh_3_)_2_]BF_4_ shows a simple *syn*‐bent to *anti*‐bent switch (like example **E4** in Scheme 1).^[8]^ (2) One of the linearly bonded nitrosyls of [RuBr(NO)_2_(PCyp_3_)_2_]BF_4_, shows a single switch to an *anti*‐bent isomer (example **E5** in Scheme 1).^[9]^ (3) The *SPY*‐5‐configured *syn*‐bent‐GS of [RuCl(NO)_2_(PCy_3_)_2_]BF_4_ switches to the *anti*‐bent isomer;^[10]^ (4) for the same substance, a switch of the *SPY*‐5 *syn*‐bent‐GS shows a single *syn*‐bent‐κ*O* switch (example **E4** in Scheme 1).^[11]^ In summary, {Ru(NO)_2_}^8^ dinitrosyls have, until now, been found with a single switchable nitrosyl ligand.^[2a]^ If the GS contains a bent nitrosyl, it is preferred as the switched ligand.

{M(NO)_2_}^10^ species possess two more electrons. If the nitrosyl ligands are of the NO^+^ type, the central metal exhibits a closed subshell with ten d‐electrons. Again, the scene for photophysics changes substantially. While all the photoinduced phenomena summarised above affect a single nitrosyl ligand, we have found, for the first time, the concerted switching of two nitrosyls at a time for the title compound of this work, dinitrosyl‐bis(triphenylphosphane)ruthenium, [Ru(NO)_2_(PPh_3_)_2_] (**1**). Moreover, since we are reporting on a double linear‐to‐bent transition of an M(NO)_2_ moiety, we have, also for the first time, the opportunity to address an everlasting arbitrariness of coordination chemistry on an experimental basis: the quest of M^*n*^ (NO^+^)/M^*n*+2^ (NO^−^) valence tautomerism in the course of an MNO linear/bent transition. (A comment on the origin of the NO^−^ formulation is given in the Supporting Information.) In the following section, we address these topics starting with the photophysical results, then including a computational analysis, and finishing with the coordination‐chemistry perspective.

## Results and Discussion

**Figure 1:**
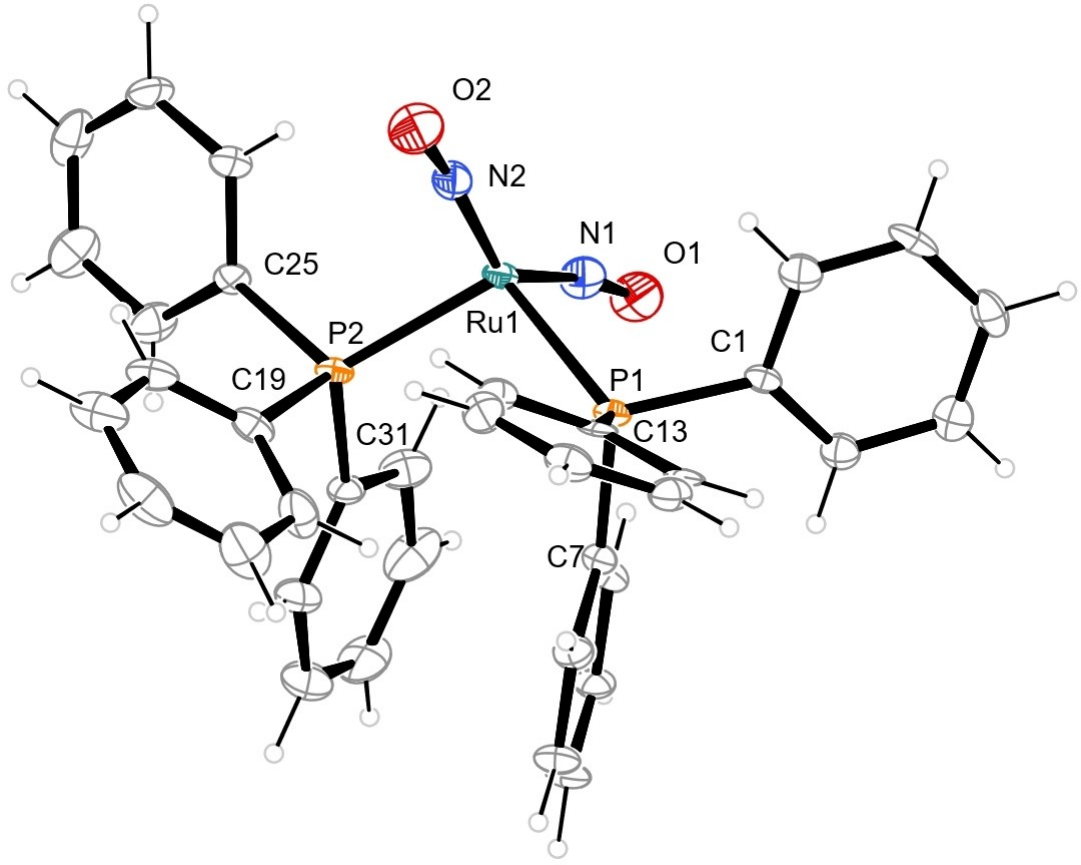
Molecules of **1** in the solvent‐free crystals, 50 % probability ellipsoids at 100 K. Distances (Å) and angles (°): Ru1−N1 1.797(5), N1−O1 1.183(7), Ru1−N2 1.802(5), N2−O2 1.177(6), Ru1−P1 2.3367(15), Ru1−P2 2.3415(15); N1−Ru1−N2 141.9(2), Ru1−N1−O1 169.9(5), Ru1−N2−O2 172.8(5), P1−Ru1−P2 104.87(5).

The preparation of the reddish, air‐stable and thermally insensitive **1** first succeeded in 1974 by the reaction of [RuH_2_(PPh_3_)_4_] with the NO^+^ source diazald (*N*‐methyl‐*N*‐nitroso‐*p*‐toluenesulfonamide, Me(NO)N‐Tos, Tos=*p*‐toluenesulfonyl) in ethanol.^[12]^ In this work, **1** was formed on attempts to introduce the hexaaquaruthenium(II) species as a multipurpose starting material for the preparation of ruthenium complexes. On treating a mixture of [Ru(H_2_O)_6_](OTos)_2_ and PPh_3_ at a molar 1 : 2 ratio in ethanolic solution with NO gas, **1** was obtained after recrystallisation in the form of solvent‐free crystals of orange to reddish brown appearance, depending on their size. (See the Experimental Section in the Supporting Information for a balanced equation.) Structure analysis showed isotypy of the crystals with those analysed by Bhaduri and Sheldrick half a century ago.^[13]^ Prior to their work, the Eisenberg and Ibers groups published the structure of the hemi‐benzene adduct **1**⋅1/2 C_6_H_6_.^[12]^ Figure 1 shows the result of our redetermination at a temperature of 100 K.

In a recent work, Ghosh and Conradie classified dinitrosyl structures in terms of electronic and geometrical parameters.^[14]^ **1** follows their rules for an {M(NO)_2_}^10^ (M=4d or 5d element) species with its large N−Ru−N angle and the “repulso” arrangement of the nitrosyls (which means that the slight deviation of the Ru−N−O function from linearity results in an outward tilt of both NO ligands). Due to the only small deviation of the RuNO angle from 180°, **1** is an example of a dinitrosyl with two linear MNO functions. For short, **1** is an (*l*‐NO‐κ*N*)_2_ species.^[5]^

**Figure 2:**
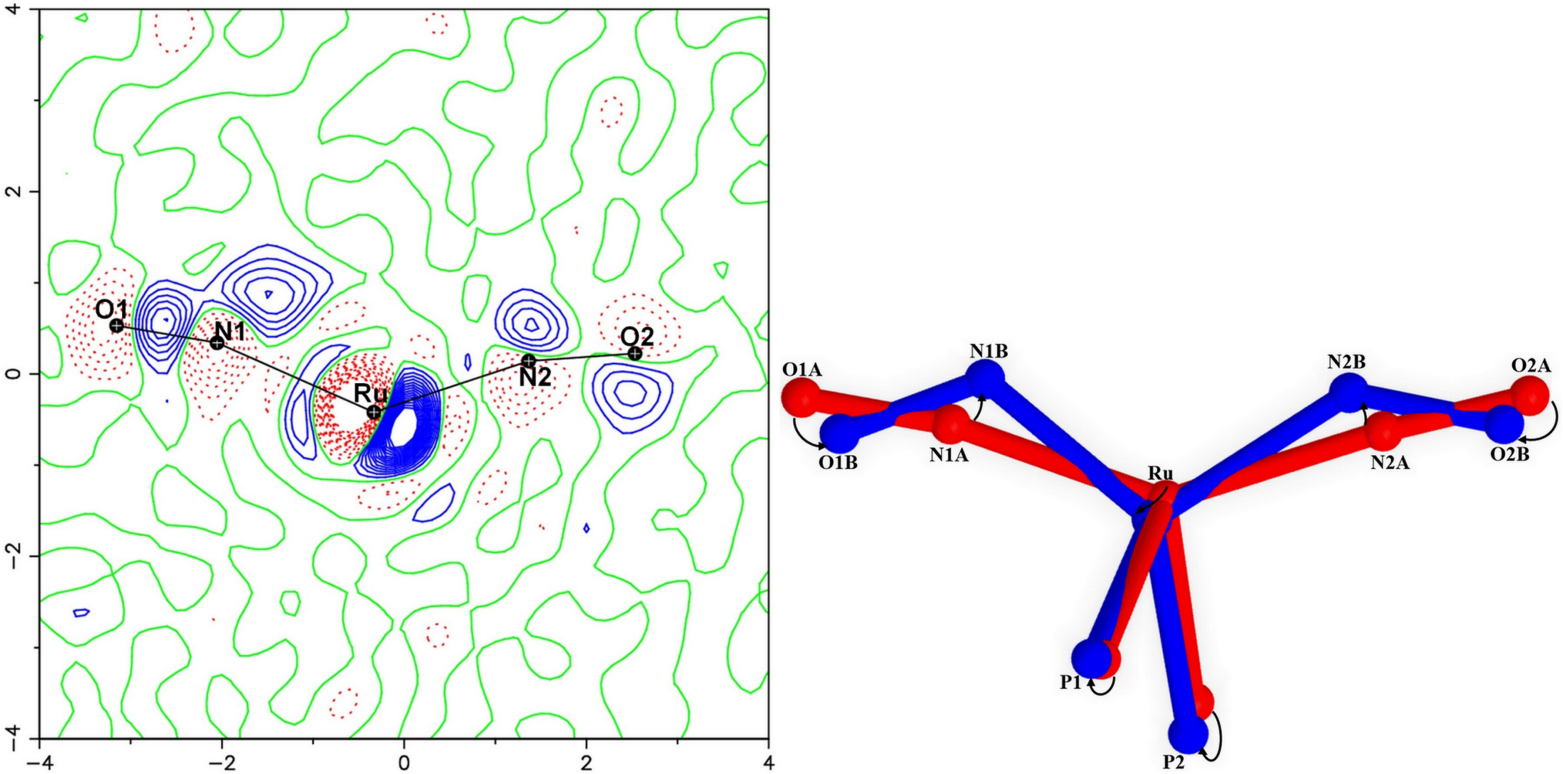
Left: Photodifference map of **1** after irradiation with 590 nm light at 100 K; blue: positive values, red: negative values; contour‐lines every 0.5 *e* Å^−3^. Right: comparison of GS and MS structure illustrating the (*l*‐NO‐κ*N*)_2_ GS to the metastable (*a*‐NO‐κ*N*)_2_ MS configurational change.

**Figure 3:**
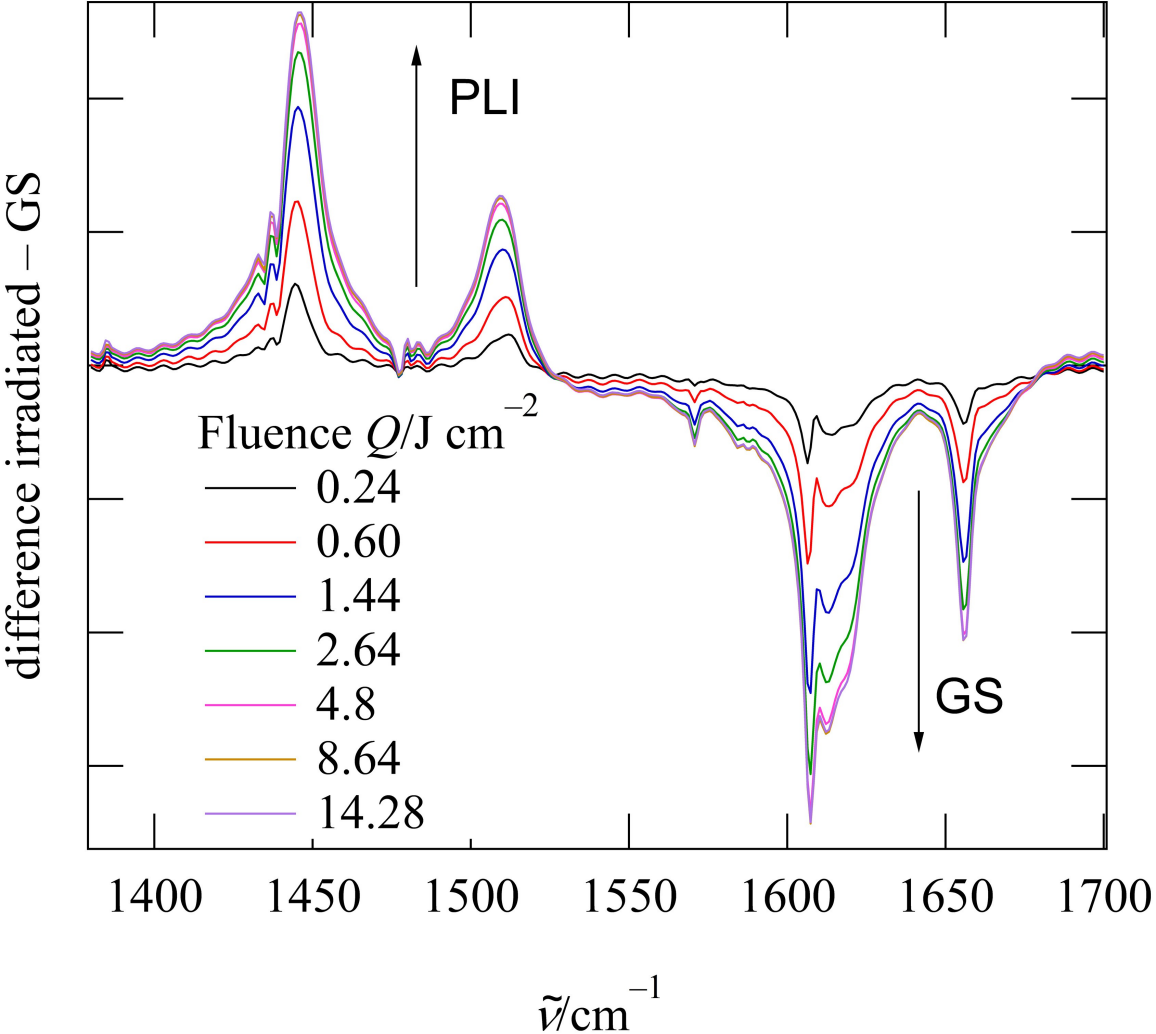
Differential infrared spectra between GS and PLI state as a function of irradiation fluence *Q*=*I t*, illustrating the conversion from GS to the PLI state.

The (*l*‐NO‐κ*N*)_2_ GS of **1** turns to the metastable (*a*‐NO‐κ*N*)_2_ double *anti*‐bent isomer upon irradiation with light in the green‐to‐orange spectral range (e.g. with 590 nm). The structure of the metastable PLI state (MS) has been determined by X‐ray photocrystallography (see the Experimental Section in the Supporting Information). Figure 2 shows the resulting photodifference map, the Fourier difference calculated between the irradiated and the non‐irradiated state in the Ru/O1/O2 plane. The map clearly highlights the slight displacement of the central Ru atom in the PLI state, as evidenced by electron‐deficient regions (red) as well as adjacent electron density accumulations (blue). In addition, major changes on both the nitrosyl ligands are observed, the GS positions being depleted (red contours) and distinct novel adjacent positions becoming visible (blue contours). From the photo‐generated electron‐density shifts one can already guess the structure of the MS, which is confirmed in the subsequent refinement: the MS with a significant occupation of 46.5 % is formed by two bent nitrosyl ligands with angles of Ru−N1B−O1B of 119.1(5)° and Ru−N2B−O2B of 135.1(4)°, much lower than in the GS (Figure 1), and a significantly reduced N−Ru−N angle of 107.4(2)° which leads, together with the slightly altered P−Ru−P angle of 106.59(2)°, to an almost undistorted tetrahedral configuration. Contrary to previously observed PLI events in dinitrosylruthenium complexes we thus find a double linkage isomer, i.e., both NO ligands undergo a significant structural rearrangement upon light irradiation. In order to confirm the existence of one PLI event in which the two NOs switch synchronously we performed infrared spectroscopy at low temperature as a function of irradiation. In the GS, the NO ligands have stretching modes associated with the bands at 1612 cm^−1^ and 1657 cm^−1^, corresponding to the asymmetric and symmetric mode (Table 1). Upon irradiation with light in the green‐yellow spectral range, these two GS bands decrease and two new bands at 1455 cm^−1^ and 1510 cm^−1^ arise. Figure 3 shows the resulting difference spectra between the irradiated sample and the GS (for complete IR spectra see the Supporting Information), measured at 100 K as a function of irradiation fluence *Q* which is the product of light intensity and irradiation time. Saturation is reached at *Q=*14 J cm^−2^, which means only two minutes of irradiation with an LED of 100 mW cm^−2^, underlining the high sensitivity. A first indication that we are dealing with a single PLI event is the fact that we observe only two new bands. Second, the decrease of both GS bands is perfectly synchronous with the increase of the two new MS bands, i.e., decrease and increase have the same time constant *Q*_0_, independent of whether we integrate the area of each single band or the sum of both. In addition, the wavelength dependence for the photogeneration of the PLI state is the same for both bands. Also the relaxation back to the GS, whether induced by red light or by heating above 230 K, affects the bands in a synchronous manner. All in all, the IR experiments strongly indicate the existence of only one PL‐isomer. The population that can be calculated from the decrease of the GS band area amounts to 50 % at its maximum found with an irradiation wavelength of 556 nm, and slightly less when using 590 nm, in agreement with the X‐ray result of 46.5 %. Additional proof was found by calorimetry. The heat released during the relaxation of the PLI state upon heating after photogeneration at low temperature can be analysed by the equation given in the Experimental Section in the Supporting Information.^[15]^ The observed signal, as shown in Figure S7 in the Supporting Information can be fitted with a single Arrhenius‐type decay, resulting in an activation energy *E*_A_ of 0.63(1) eV and a frequency factor *Z* of 8.0(3)⋅10^11^ s^−1^. The total released enthalpy *H*_tot_ depends on the population within the sample (which depends on the penetration depth) and the energy of the metastable state. Using the computed instability of the MS of approximately 0.6 eV, an estimate of the population of the PLI state in the calorimetric setup is 15–20 %.

**Table 1:** Isomer energies, relative to *E*(GS)=0, and the calculated energies of the N−O stretches on the BP86/def2‐TZVP+D3 level of theory (analytical frequencies with Orca5). More or less decoupling of the symmetric/asymmetric N−O stretches is covered by footnotes. For the MS2‐type NO‐κ^2^ *N*,*O* isomers, the atom with the shorter contact to Ru is underlined. For an extended version of the table with more starting variants, see Table S3 in the Supporting Information.

	*E*/eV	*E*/kJ mol^−1^	$\tilde{\nu }$_sym_/cm^−1^	$\tilde{\nu }$_asym_/cm^−1^
**experiment**:				
(*l*‐NO‐κ*N*)_2_	0	0	1657	1612
(*a*‐NO‐κ*N*)_2_	–	–	1510	1445
**(NO‐κ*N*)_2_ isomers**:				
(*l*‐NO‐κ*N*)_2_	0	0	1709	1676
(*a*‐NO‐κ*N*)_2_	0.631	60.9	1553	1519
(*s*‐NO‐κ*N*)_2_	1.180	113.8	1508	1463
**(NO‐κ*N*)(NO‐κ*O*) isomer**:				
(*l*‐NO‐κ*N*)(*l*‐NO‐κ*O*)	1.975	190.6	1672^[a]^	1619^[b]^
**(NO‐κ*O*)_2_ isomers**:				
(*l*‐NO‐κ*O*)_2_	4.122	397.7	1607	1591
(*a*‐NO‐κ*O*)_2_	4.355	420.2	1575	1547
**(*l*‐NO)(NO‐κ^2^** ***N***, ***O*** **) isomers**:				
(*l*‐NO‐κ*N*)(NO‐κ^2^ *N*,*O*)	1.135	109.5	1661^[c]^	1282^[d]^
(*l*‐NO‐κ*N*)(NO‐κ^2^ *N*, *O*)	2.205	212.7	1677^[c]^	1395^[d]^
(*l*‐NO‐κ*O*)(NO‐κ^2^ *N*,*O*)	3.133	302.3	1631^[e]^	1265^[d]^

[a] largely NO‐κ*N*, [b] largely NO‐κ*O*, [c] *l*‐NO‐κ*N*, [d] NO‐κ2*N*,*O*, [e] *l*‐NO‐κ*O*.

To investigate the conceivable isomers of **1**, the energetic landscape was scanned on the BP86/def2‐TZVP+D3 level of theory (Orca, versions 4.2.1 to 5.0.3).^[16]^ Minima were verified by means of frequency analyses. Table 1 summarises significant local minima. Due to their relationship with the experimentally detected PLI state, the NO‐κ*N*‐bonded isomers are of special interest. As a result, the found PLI state, with its double *anti*‐bent‐bonding, is the most stable of the non‐GS isomers. The related double *syn*‐bent isomer is a local minimum as well but of markedly less stability. Of special interest for the discussion of the isomerisation mechanism below is the fate of an (*a*‐NO‐κ*N*)(*s*‐NO‐κ*N*) form. This mixed *a*/*s*‐bent isomer is unstable on the chosen level of theory and relaxes to the (*l*‐NO‐κ*N*)_2_ GS (Table S3 in the Supporting Information).

For an in‐depth investigation of the GS and the experimentally detected PLI state, various levels of theory were checked for the GS (see Table S4 in the Supporting Information). As a result, the BP86/def2‐TZVP+D3+CPCM(toluene) level was used for the subsequent computations. Toluene as the solvent for an approximation to the data of the solvent‐free solids was chosen since the X‐ray analyses of both isomers showed that the phenyl groups of the PPh_3_ ligands of neighbouring molecules mediate the intermolecular contacts (Figures S3 and S5 in the Supporting Information show these contacts). With this approach, we obtained the values of Table 2 for the (*l*‐NO‐κ*N*)_2_ GS and the (*a*‐NO‐κ*N*)_2_ MS.

**Table 2:** Mean distances and angles along the path GS‐TS‐MS. Calculated metrical and spectroscopic values from a BP86/def2‐TZVP+D3+CPCM(toluene) approach, energies include a ZORA correction (see Table S5 in the Supporting Information). Note the limited precision of the MS_exp_ metrical values due to the ≈1 : 1 disorder of GS and MS in the photocrystallographic experiment.

	GS_exp_	GS_calc_	TS_exp_	TS_calc_	MS_exp_	MS_calc_
	(*l*‐NO‐κ*N*)_2_				(*a*‐NO‐κ*N*)_2_	
Ru−N/Å	1.800	1.795		1.868	1.784	1.829
N−O/Å	1.180	1.193		1.190	1.237	1.209
Ru−N−O/°	171.4	173.9		141.5	127.1	127.8
N−Ru−N/°	141.9	140.7		111.5	107.4	105.5
$\tilde{\nu }$_sym_/cm^−1^	1657	1678		1615	1510	1521
$\tilde{\nu }$_asym_/cm^−1^	1612	1632		1557	1445	1476
*E*/eV		0		1.240		0.611
*E*/kJ mol^−1^		0		119.6		59.0
*E*_a_/eV			0.63(1)	0.628		
*E*_a_/kJ mol^−1^			61(1)	60.6		

**Figure 4:**
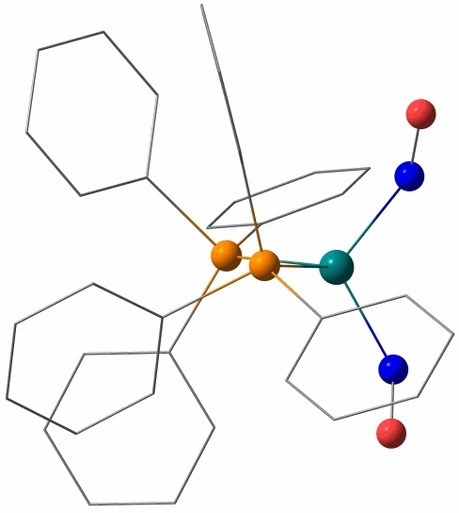
The transition state (TS) which connects the double‐linear GS with the double‐bent MS of **1**. The TS's strongly negative vibrational mode (−835 cm^−1^) traces the decay path if the photo‐generated MS is warmed above 230 K (see Supporting Information for an animation of the movement).

Table 2 also includes the parameters of the transition state (TS) between the GS and the MS which was consistently computed by various approaches. Figure 4 shows the structure of the TS. An animation of the vibration (see Supporting Information) with the most negative frequency of −835 cm^−1^ confirms the argumentation of the photophysical section by showing a concerted motion of both nitrosyl ligands. (The two rightmost phenyl groups of Figure 4 follow the nitrosyls’ motion with a markedly lower energy gain of −32 cm^−1^ and −23 cm^−1^.) Moreover, it is the entire Ru(NO)_2_ moiety including the Ru atom which swings between the states—in agreement with the photodifference map in Figure 2. Certainty that we have in fact detected the correct TS comes from another experimental method, the above‐mentioned DSC investigation. The calculated *E*_a_ entries in Table 2, the difference between the TS and the MS energies, are close to the measured activation wall of the decay of the MS.

**Figure 5:**
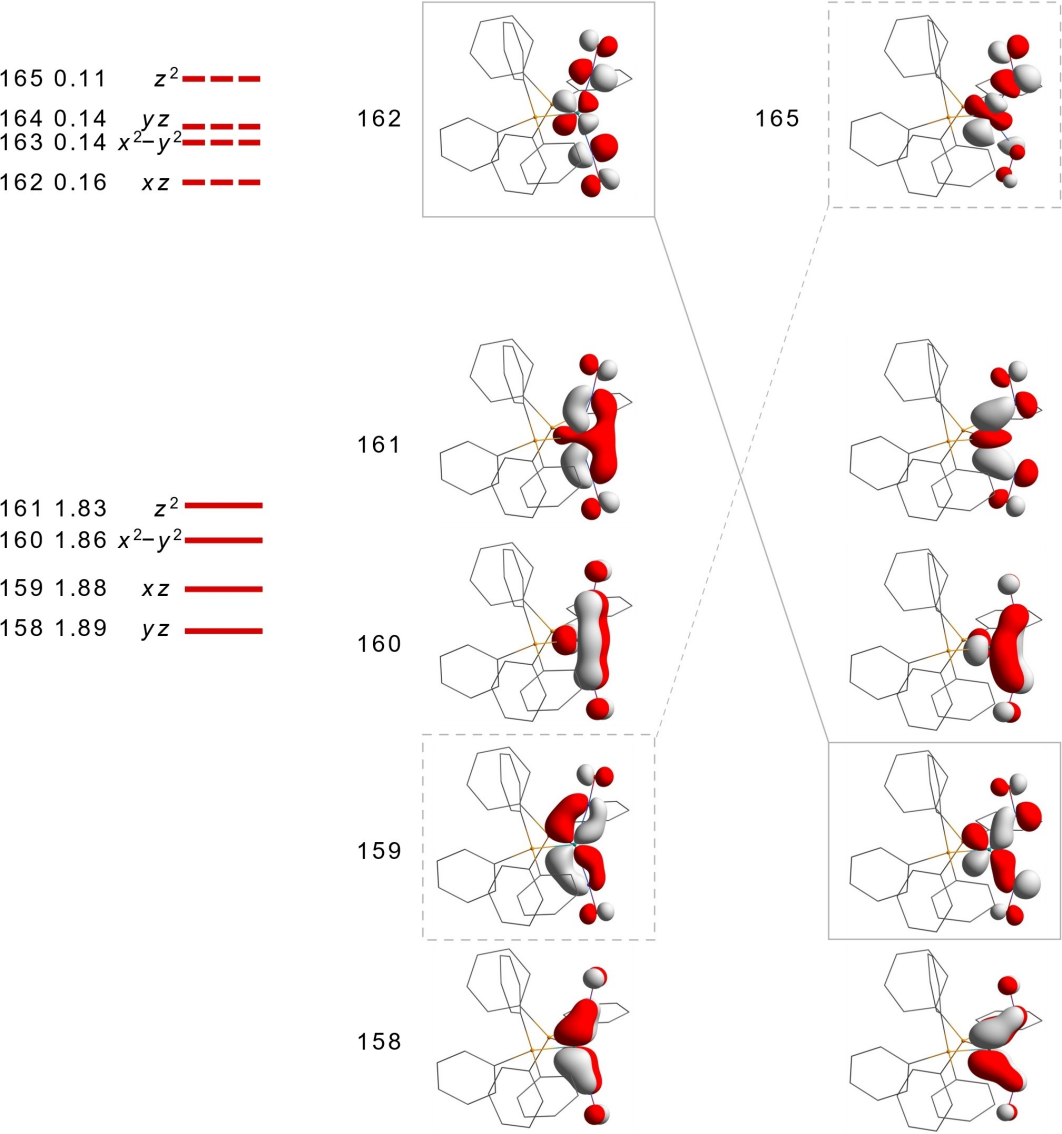
From bottom to top: the four orbitals that are doubly occupied in the major configuration of the GS (left) and the MS (right) in a CASSCF(8,8) approach (weight of the major 22220000 configuration in both the GS and MS: 0.76; leftmost: MO numbers, occupation and assignment with *z* up, *x* right and *y* into the plane for the GS; almost the same values were obtained for the MS). The topmost MOs are empty in the major configuration; three more antibonding MOs of the active space are skipped.

The electronic origin of the characteristics of the radiationless MS‐to‐GS transition, in particular the concerted nitrosyl motion, becomes evident from a look at the changes of the frontier MOs. Figure 5 shows the correspondence of the significant orbitals in terms of a CASSCF(8,8) approach with a small active space. (At the Ru atom, the nonbonding 4d_*xy*_ orbital was left out; in a CASSCF(9,10) approach, its occupation was 1.99.) At first glance, we see for three of the four doubly occupied orbitals a completely conservative change. Without changing the symmetry of the orbitals’ interaction, the nitrosyl ligand rotates (from GS to MS: the upper one counterclockwise, the lower one clockwise; “disrotatory” in the sense of IUPAC's Gold‐Book definitions of electrocyclic reactions) from the linear to the bent position (MOs 158, 160, 161). MO159 is an exception. If the nitrosyls perform their disrotatory motion in MO159 with its Ru‐4d_*xz*_ contribution, the bond in the GS turns to an antibond in the MS and *vice versa*.

As a consequence, the Ru‐4d_*xz*_ orbital is a largely metal‐only MO in the TS. The loss of a backbond in the TS can be traced in terms of electron densities. Using QTAIM charges (see below) as a measure, we find, both in the GS and the MS, mean charges of the two nitrosyls of −0.54 *e* and −0.49 *e*, respectively, whereas the absolute value of the charge decreases in the TS and reaches −0.40 *e*. It is this balance of a symmetry‐allowed transition of three out of four metal d‐orbitals and a symmetry‐forbidden transition of the fourth that we see as the origin of the transition's characteristics, specifically the radiation‐less decay of the MS. On the one hand, the conservative part assures a not too high energetic wall towards the PLI state. On the other hand, there is in fact an activation barrier which allows the isolation of the metastable isomer. The latter point makes a difference to most {MNO}^8^ species which lack this barrier (see above the platinum example). A closer look at the conservative part unravels another decisive prerequisite for the symmetry‐allowed motion of the nitrosyls: their turn has to be synchronous to keep track on a low‐energy path of the hypersurface.

The loss of a Ru−NO bond in the TS matches with the tentative mechanism of the GS‐to‐MS transition, initiated by the excitation by green light, the complementary colour of the reddish crystals of **1**. A TDDFT approach on the BP86/def2‐TZVP+D3+CPCM(toluene) level showed the electronic origin of the colour. Starting with the HOMO→LUMO excitation as the lowest‐energy transition (522 nm, 2.376 eV, 19 163 cm^−1^), also the next excitations in the visible part of the spectrum resemble the transition of one electron from the Ru−NO bonding to the Ru−NO antibonding regime (Figure 5, left), thus eliminating one of the bonds. Illumination of samples of **1** thus causes the loss of one Ru−NO‐backbond‐related MO which initiates the motion of the nitrosyl ligands.

The formation of the metastable isomer from the GS is accompanied by an IR redshift of the NO stretching modes of about 150 cm^−1^. The shift is less than that of a GS‐to‐MS2 excitation in {MNO}^≤6^ species but is typical if linear and bent forms of related nitrosyls are compared (see Ref.^[17]^ for {CoNO}^8^ examples). The observation of a redshift of this magnitude on the bent side appears to be a major reason for a custom which emerged shortly after the discovery of bent‐bonded nitrosyls, namely to assign an NO^+^ ligand in a linear MNO unit but a singlet‐NO^−^ ligand in a bent one.^[18]^ In terms of the Dewar–Chatt–Duncanson (DCD) model, a higher negative charge of an acceptor ligand indicates the increasing strength of M→L backbonds by an increasing population of antibonding ligand‐MOs. If this common interpretation would be accepted for the isomers of this work, the double‐linear‐to‐double‐bent transition of **1** would be a formal 4‐electron redox process which connects two valence tautomers with Ru^−II^/(NO^+^)_2_ and Ru^II^/(NO^−^)_2_ cores. However, recent work has collected arguments that a nitrosyl ligand's oxidation state (OS) needs some kind of justification and must not be deduced simply from the M−N−O angle (in full agreement with an early warning by Enemark and Feltham).[^17^, ^19^] Now, the GS/MS couple of **1** provides, for the first time, an experimentally detected isomeric bent/linear couple which allow us to check the charge issue. Two methods are used to tackle this problem. The first is the EOS (effective oxidation state) method introduced by the Salvador group on the basis of QTAIM values to decide the Ru^−II^/(NO^+^)_2_ vs. Ru^II^/(NO^−^)_2_ ambiguity.^[20]^ Second, we use the local‐mode analysis by Cremer and Kraka to disentangle charge and bond‐strengths arguments.^[21]^

Table 3 shows the change of relevant parameters along the GS‐TS‐MS transition. Notably, the 2‐electron‐5‐centre picture of four occupied canonical frontier MOs is replaced by parameters that describe the two individual Ru−NO interactions. Since the three states deviate from *C*_2_‐symmetry (GS and MS slightly, and TS markedly), we argue with mean values here but give individual numbers in Table S6 in the Supporting Information. The first entry of Table 3 shows the charge of the nitrosyls (Ru and phosphane charges are listed in Table S7 in the Supporting Information) which have already been discussed above. Most notably for the NO^+^/NO^−^ issue: the bent‐bonded nitrosyls do not bear a higher negative charge.

**Table 3:** QTAIM, EOS and local‐mode analysis of the isomers of **1** and the transition state that connects them. *Q* is the QTAIM charge, EOS is the effective oxidation state, *R* is defined in Ref. [20c], λ‐values are occupations of the nitrosyl's localised effective fragment orbitals (EFOs) with electron pairs (ip=in plane, oop=out of plane). Local‐mode analysis contributed the last two lines with *k* ^a^ as the local force constant. The required wave‐function files and Hessians are computed on the BP86/zora‐def2‐TZVP+ZORA+D3+CPCM(toluene) level. The table contains mean values; individual values of the nitrosyls are compiled in Table S6 in the Supporting Information.

	GS	TS	MS
*Q*_NO_/*e*	−0.54	−0.40	−0.49
EOS(Ru)	−2	−2	−2
EOS(NO)	+1	+1	+1
*R* in %	57.2	53.9	63.5
*λ* ^NO^(σ‐donor)	0.86	0.90	0.91
*λ* ^NO^(ip‐π*‐acceptor)	0.44	0.48	0.41
*λ* ^NO^(oop‐π*‐acceptor)	0.42	0.26	0.38
*k* ^a^(Ru−N)/N cm^−1^	4.37	2.51	2.73
*k* ^a^(N−O)/N cm^−1^	10.80	10.47	9.17

The next six lines show the result of the EOS analysis. In this approach, the electron density is fragmented by a suitable method, here QTAIM, and expressed in effective fragment orbitals (EFOs). To determine the fragment's OS, the occupation *λ* of the EFOs is compared with the occupation of the other EFOs and, in the sense of the OS as a winner‐take‐all principle, the respective electron (pair) of a bond is assigned to the fragment with the highest occupation. A value *R* is defined to indicate borderline situations.^[20c]^ *R=*100 % indicates a highly polar or ionic bond, whereas *R=*50 % as a worst‐case value for an OS assignment indicates perfect covalency. Nitrosyl‐metal bonds tend to approach this 50 % border. However, though close to a nonpolar covalent bond, linear as well as bent nitrosyls which were analysed by the EOS procedure have been assigned, as a rule, NO^+^ based on typical R values around 60 %.^[17]^ **1** is no exception to this rule. Both isomers of **1**, the linear but also the bent one, arise to be d^10^‐Ru^−II^(NO^+^)_2_ species. The occupations *λ* show the individual contributions of the three interactions of M−NO bonding that has to be taken into consideration. *λ* ^NO^(σ‐donor), the occupation of mainly the lone pair at the nitrogen atom, belongs to a markedly weak donor. In the GS, with an occupation of 0.86, only little electron density was transferred to initially empty metal orbitals (5s, 5p). This small amount is still less in the bent MS where the N‐centered lone pair points past the central metal.

On the contrary, as expected for a strong acceptor ligand, the two interactions of the initially empty NO^+^(π*) with filled Ru orbitals are decisive (with respect to the plane defined by the five atoms of the dinitrosylruthenium core, we distinguish “in‐plane” and “out‐of‐plane” interactions). The occupations of these nitrosyl‐π* MOs are close to 0.4 whereas the corresponding occupations of the metal orbitals are around 0.6. These numbers, which approach the 0.5:0.5 ratio of an ideal covalent bond, are the basis for the assignment of the oxidation states. With the metal's share being slightly larger, the electron pairs of both interactions go to the metal on OS determination which leaves the ligand as NO^+^. More importantly: despite the N−O stretch's redshift from GS to MS, charge transfer into the NO‐π* MOs is less for the bent MS in terms of the *λ* values.

The NO^+^ assignment may be irritating if we have a look at the nitrosyl ligand's “real” negative charge *Q* of approximately −0.5 *e* as the result of the marked electron‐density gain into the NO‐π* MOs. However, we have to take into account that an oxidation state is the nearest integer of the “real” charge only if a single bond is considered. In the double‐backbond scenario of nitrosyl bonding, balancing the electron flow mirrored by the λ values clarifies the point. Starting with an NO^+^, the ligand's real charge is the sum of the small charge depletion from the σ‐donor orbital and the substantial charge influx into the N−O antibonds. Thus, the nitrosyls’ initially empty acceptor orbitals are occupied by ≈0.4 electron pairs per NO‐π*, i.e., with ≈0.8 electron pairs per nitrosyl ligand. The nitrosyl's weak donor ability caused a little electron loss of roughly 0.1 electron pairs from the σ‐donor orbital as indicated by the remaining ≈0.9 electron pairs as the lone‐pair occupation. Substracting the σ‐donor loss of 0.1 electron pair we are left, in total, with the transfer of 0.7 electron pairs to the initial NO^+^. In terms of charge, 0.7 of an electron pair resembles −1.4 elementary charges *e*. The initial charge +1 *e* of an NO^+^ thus turns to −0.4 *e* in this in/out balance, in good agreement with the nitrosyl charge of the QTAIM analysis. It should be noted at this point that the customary formulation of a bent‐bonded nitrosyl as NO^−^ would require that *λ* ^NO^ has to exceed *λ* ^M^ for one and only one of the bonds, usually assumed (though without justification) for the in‐plane M−NO σ‐bond.

The last two lines of Table 3 list local force constants as a measure of bond strengths which enables us to clarify the apparent inconsistency of the N−O stretch's redshift despite the lack of an additional electron‐density transfer. In terms of force constants, a linear‐to‐bent transition in fact weakens the N−O bond. Contrary to the DCD model, however, the Ru−N bond is also weakened. Hence, the GS/MS transition of **1** is a non‐DCD event which lacks the DCD‐typical charge/bond‐strength correlation of an acceptor ligand. The physical origin of the observed redshift, lateral electrostatic influence on bending, is addressed in a separate work.^[22]^

The TS is worth a closer look. With *λ=*0.48, the in‐plane‐NO‐π* orbital accepts the highest amount of electron density through the Ru−NO in‐plane bond. It is thus the out‐of‐plane Ru−NO π‐bond with an occupancy of only 0.26 which is responsible for the Ru−NO bond weakening, indicated by the local Ru−N force constant, at the highest point of the transition.

## Conclusion

In conclusion, we found, for the first time, a synchronous photo‐induced linkage isomerisation of two nitrosyl ligands of a dinitrosyl complex. Due to the ≈50 % population of the metastable (up to 230 K) excited state, reliable experimental verification of the MS succeeded and showed the formation of a double *anti*‐bent, κ*N*‐bonded MS from a double‐linear GS. Specifically, the synchronicity of the MS's formation and decay is without precedence in PLI research. Computation unravelled the origin of the synchronous transformation. Only the synchronous disrotatory motion of the pair of nitrosyl ligands, pictorially the *pas de deux* of a nitrosyl couple, allows a smooth transition free of too many bond ruptures. Specifically, two factors are decisive for the transition path (which should have physical reality for the radiationless decay of the MS). The first is a symmetry‐conserving MO motion for most frontier orbitals. In electron‐rich {MNO}^*n*^ species, this fact is common and usually prohibits an activation barrier between bent and linear isomers, thus leaving only the more stable of the two “ground‐state candidates” isolable. As a peculiarity of **1** and as a second factor, one of the metal's d orbitals experiences a bond‐to‐antibond transition on the motion of the two nitrosyls with the necessity to break *one* Ru−NO bond in the transition state. As a result, there *is* an activation barrier that makes both isomers tangible. Moreover, due to the symmetry‐conserving first factor, the motion has to be synchronous and disrotatory. This type of motion might find application in molecular motors, since low doses of yellow light can switch it on, inducing a movement in one direction.

Contrary to the usual charge assignment in linear and bent MNO functions, the oxidation states (OSs) as well as the nitrosyls’ charge do not change significantly in the course of the linear/bent transition which leaves the OSs unaltered at the Ru^−II^(NO^+^)_2_ level. Hence, there is no justification to assign NO^+^ ligands to the linear but NO^−^ to the bent isomer. Notably, the GS/MS couple of **1** allows us to check this long‐standing NO^+^/NO^−^ issue on a couple of experimentally verified isomers.

## Supporting Information

In the Supporting Information, we provide an Experimental Section, a typical M−N−O scan of an {MNO}^8^ system, a comment on NO^+^/NO^−^ tautomerism, details of the applied methods, and an animation showing the concerted motion of both nitrosyl ligands.

## Conflict of interest

The authors declare no conflict of interest.

1

## Supporting information

As a service to our authors and readers, this journal provides supporting information supplied by the authors. Such materials are peer reviewed and may be re‐organized for online delivery, but are not copy‐edited or typeset. Technical support issues arising from supporting information (other than missing files) should be addressed to the authors.

Supporting Information

Supporting Information

## Data Availability

The data that support the findings of this study are available from the corresponding author upon reasonable request.
