# Supplementary material for: Pas de Deux of an NO Couple: Synchronous Photoswitching from a Double‐Linear to a Double‐Bent Ru(NO)2 Core under Nitrosyl Charge Conservation
Source: Angew Chem Int Ed Engl. 2022 Sep 15;61(42):e202210671. doi: 10.1002/anie.202210671 (PMC9826364; doi:10.1002/anie.202210671)
Supplement: Supplementary file 1 — Supporting Information [file ANIE-61-0-s002.pdf]

Angewandte Chemie
Eine Zeitschrift der Gesellschaft Deutscher Chemiker

Supporting Information

# *Pas de Deux* of an NO Couple: Synchronous Photoswitching from a Double-Linear to a Double-Bent $Ru(NO)_2$ Core under Nitrosyl Charge Conservation

*A. Hasil, D. Beck, D. Schröder, S. Pillet, E. Wenger, T. Woike, P. Klüfers*, D. Schaniel**

# Supporting Information

## Experimental Section

### Synthesis of 1

**1** was formed on attempts to use hexaaquaruthenium(II) tosylate, $[Ru(H_2O)_6](OTos)_2$ (Tos = *p*-toluenesulfonyl, $MeC_6H_4SO_2$), as an educt for the preparation of nitrosyl-phosphane-ruthenium compounds.[1] A solution of the aqua complex in ethanol was treated with triphenylphosphane and, subsequently, with NO gas. **1** formed according to:

$$[Ru(H_2O)_6](OTos)_2 + 2PPh_3 + 4NO \rightarrow \mathbf{1} + 6H_2O + 2NO^+ + 2OTos^-$$

The formal product $NO^+$ would react to ONOEt in pure ethanol as the solvent. Since water is liberated in the course of the reaction, $HNO_2$ and/or its decomposition products might be formed as well. We have not investigated this point further.

### Differential scanning calorimetry (DSC)

The DSC experiments were performed on a DSC1 Mettler-Toledo instrument equipped with a high sensitivity DSC HSS8 sensor. The samples ($m$ = 1.71 mg of the powder) were homogeneously spread in a standard aluminium crucible (40 μL) such that it formed a thin layer covering the whole surface of the crucible. Irradiation of the sample was performed at 150 K through a glass window, using Laser light at 556 nm with light intensity of 30 mW cm$^{-2}$ for 20 min. For the detection of the enthalpy release during thermal relaxation of the photoinduced states, the sample was then heated from 150 K to 300 K at a heating rate of $\beta$ = 4 K min$^{-1}$. As a reference measurement the sample was measured using the same protocol but without light irradiation. The difference between irradiated and non-irradiated heatflow was evaluated using the equation

$$\frac{\mathrm{d}H(t)}{\mathrm{d}t} = \Delta H_{\mathrm{tot}} \mathrm{Z} \exp\left( -\frac{E_\mathrm{A}}{k_\mathrm{B}T} - \frac{Z}{\beta} \int_{T_0}^{T} \exp\left(-\frac{E_\mathrm{A}}{k_\mathrm{B}T'}\right) \mathrm{d}T' \right)$$

which assumes an Arrhenius-like behavior with $E_\mathrm{A}$ the activation energy and $Z$ the frequency factor.[2] $H_{\mathrm{tot}}$ denotes the total enthalpy released during the decay and $k_\mathrm{B}$ is the Boltzmann constant.

### Infrared and UV/Vis spectroscopy

IR measurements were performed at different temperatures between $T$ = 10 and 300 K and the sample was kept in a vacuum inside a closed-cycle cryostat, using a Nicolet 5700 FT-IR spectrometer with a resolution of 2 cm$^{-1}$. The sample was ground, mixed with KBr and pressed into pellets. The KBr pellets were bonded onto the cold finger of the cryostat using silver paste, and irradiated through a KBr windows with LED light in the wavelength range of 365 – 735 nm. The maximum population of about 50% was achieved by irradiation of the sample with light of $\lambda$ = 530 – 590 nm. UV/Vis spectroscopy were performed using a CARY 4000 spectrometer in the wavelength range 900-350 nm with a resolution of 2 nm. Sample preparation was as for IR experiments and temperature was 100 K in the same cryostat as used for IR experiments.

## Photocrystallography

Single-crystal X-ray diffraction data were collected on a SuperNova microfocus-source diffractometer equipped with an ATLAS CCD detector, using MoK$_\alpha$ radiation ($\lambda$ = 0.71073 Å) and an Oxford Instruments nitrogen-flow cryostream was used to keep the temperature at 100 K. A single crystal was mounted on a micro-gripper loop using vacuum grease. In a first step, diffraction data were collected at 100 K in the ground state. The unit-cell determination and the data reduction were performed using the CrysAlisPRO program (Oxford Diffraction Ltd, Yarnton, Oxfordshire, England, 2011) on the full data set. 390552 reflections were measured up to a maximum resolution of sinθ $\lambda^{-1}$ = 0.997 Å$^{-1}$ and merged to 25703 unique reflections ($R_{\text{int}}$ = 0.055). A numerical absorption correction was performed. The corresponding structure was solved in the space group $P\,2_1/n$ by direct methods using the SHELXS program and refined on $F^2$ by weighted full matrix least-squares methods using the SHELXL program (version 2018).[3] All non-H atoms were refined anisotropically.

Another single crystal was mounted on a glass fiber using vacuum grease. The sample was then irradiated at 100 K with a laser diode of 590 nm ($P$ = 1.6 mW) for 60 min in the complete dark, until the photo-stationary state was reached, the sample being continuously rotated during the irradiation. According to the infrared results, at this temperature and irradiation wavelength, the metastable PL-state can be selectively populated with a sufficiently high population to enable a meaningful photocrystallographic experiment. It is noteworthy that due to high photosensitivity of the sample, illumination was carried out in the absolute darkness in order to get the highest possible MS population. Complete diffraction data were collected in the photo-stationary state; no space-group change occurs with respect to the ground state. The unit-cell parameters change only slightly from $a$ = 9.22200(11) Å, $b$ =36.4379(4) Å and $c$ = 9.90412(12) Å in GS to $a$ = 9.2775(5) Å, $b$ = 36.4176(16) Å and $c$ = 9.9290(5) Å in the photo-induced state. The angles only slightly change from $\beta$ = 111.0381(13)° in the GS to $\beta$ = 111.867(6)° in photo-induced state. The unit-cell volume expands from 3106.24(7) Å$^3$ in the GS to 3113.3(3) Å$^3$ in the photo-induced state. 36255 reflections were measured up to a maximum resolution of sinθ $\lambda^{-1}$ = 0.759 Å$^{-1}$, and merged to 10613 unique reflections ($R_{\text{int}}$= 0.046). Numerical absorption correction was performed. Relevant data are given in Table S1.

Photo-difference maps (Figure 2) were calculated for visualization of the light-induced changes in electron density, and for identification of the related structural changes from the GS to the 590 nm photo-irradiated state. Common independent reflections between the GS and the photo-irradiated state were used to compute the experimental X-ray photo-difference map by Fourier transform of the difference $[F_o^{\text{photo-irradiated}}(hkl) - F_o^{\text{GS}}(hkl)]$, using the structure factor phases from the GS structural refinement. 10348 common independent reflections were included in the calculation.

## Computational Studies

Structure optimizations and analytical frequency analyses of all species were performed by Orca, versions 4.2.1 to 5.0.3,[4] using the Karlsruhe Def2 basis sets,[5] their auxiliary basis Def2/J,[6] various density functionals, the Becke-Johnson-damped D3 dispersion correction,[7] and the integration acceleration method RI.[8] Local-mode analysis was performed by LmodeA 2.0.0 (W. Zou, Y. Tao, M. Freindorf, M. Makos, N. Verma, E. Kraka, Dallas 2020) after adapting the input routine to Orca 4/5 Hessians. QTAIM analyses were performed using MultiWFN 3.8.[9] The converged wave functions of the ORCA calculations were converted via orca_2aim from their gbw files to MultiWFN-compatible wfn files. After export to the FCHK format, the wfn file was passed to the APOST-3D 4.0 program,[10] together with an input file which specified the fragmentation, yielding the effective fragment orbital (EFO) occupations and finally the oxidation state of each fragment. The background of EOS-related computations in the field of nitrosyl complexes is given in the Experimental Section of Ref. [11], in particular the definition of $R$.

### The Pt–N–O scan mentioned in the Introduction

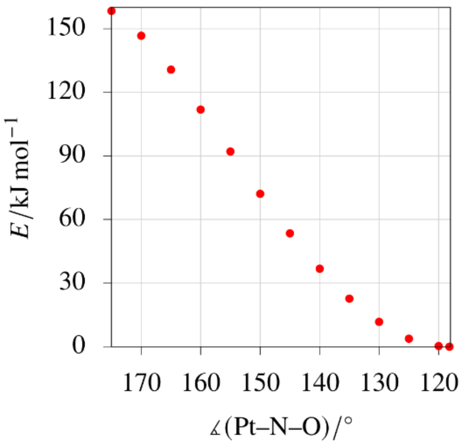


**Figure S1.** Energy rise on linearising the bent PtNO ground state in $[PtCl(NH_3)_4(NO)]^{2+}$; BP86/def2-TZVP+D3+CPCM(∞), Pt–N–O at minimum: 118.173°.

### Comment on $NO^+$/$NO^-$ valence tautomerism

The related $\{CoNO\}^8$ compound $[CoCl_2(NO)(PMePh_2)_2]$ provides a recent example to introduce a key issue concerning nitrosyl bonding modes: can bent and linear MNO fragments be addressed as valence tautomers?[12] A statement made in Ref. [12] regarding linear/bent transitions of the cobalt compound, inline with a IUPAC comment, shows the current perception: "The oxidation states of the Co are nominally +I … and +III … . Structural isomerization is therefore accompanied by a change in the formal oxidation state of the Co atom, and the isomers are valence tautomers".[13] This statement stands and falls with the assignment of oxidation states. If and only if the bent and linear structures go along with an oxidation-state change, there will be valence tautomers and a 2-electron redox process will transform them. Recent work on $\{CoNO\}^8$ species has shown that bent CoNO units are compatible with a $Co^I/NO^+$ OS assignment. Moreover, a tentative linear isomer of the real bent species $[Co(fpin)_2(NO)]^{2-}$ has been found as a local energetic minimum.[14] The $[Co(fpin)_2(NO)]^{2-}$ ion is not a single exceptional case. About 20 mostly bent and few linear CoNO moieties of various $\{CoNO\}^8$ species were analysed in a recent work.[11] In fact, $Co^I/NO^+$ appears as the standard assignment both for the linear but also for the bent species in the course of a wavefunction-based OS assignment, Salvador's EOS procedure.[15]

Such a computationally supported determination of both an OS as well as the 'real' charge of an NO ligand in the sense of electron density analysis, both in terms of experiment and computation, appears to have not attracted interest in the past. Instead, the equality 'bent = $NO^-$' entered the chemical literature at a moment's notice, without any justification. While the first unambiguous proof of a bent MNO fragment by Hodgson and Ibers in 1968 came without an interpretation of the nitrosyl's charge, already the preliminary report of a second X-ray structure on the $\{CoNO\}^8$ species $[CoCl(en)_2(NO)]ClO_4$ by Snyder and Weaver a year later contains the $NO^-$ formulation. Though Enemark and Feltham, in their 1974 review, warned to derive a charge from a bonding mode, since then the bent-MNO/$NO^-$ equality is found as a self-evidence that does not need a proof.[16]

Missing an explicit justification may imply that the $NO^-$ can be derived from established rules of chemistry. As a particularly suggestive tool in favour of the $NO^-$ formulation, we find Lewis formulae. There, the free singlet $NO^-$ ligand with a double bond between N and O is drawn and two lone pairs are attached to each atom in an $sp^2$-type manner. One of the nitrogen's lone pairs then is used to formulate a bond to M. The assignment of a −I OS to NO means that this bonding pair keeps a higher share at the NO and a lower at M. An analoguous formulation applies to a nitrito-κ*N* ligand for example. As a 'pro' of this formulation, we have to deal with one bond only, the σ-M–NO bond, not with the two equivalent π-bonds of a linear M–NO function. If the latter bonds are counted in favour of M on OS assignment, an $NO^{3-}$ ligand would result which may make an author feel uneasy.

The ‘con’ of the eventually arbitrary $NO^-$ assignment is clear. Why not determine the M–NO polarity by a suitable quantitative method? Computational methods such as Salvador’s EOS or Head-Gordon’s LOBA procedures are available. Bond for bond, the electron pairs (separate spins for paramagnets) are allocated to the bonding partner with the higher share resulting in the OS after summation.[15a] In fact, established assignments are confirmed such as the OS of a κ*N*-bonded nitrito ligand as −I. However, bent-bonded nitrosyl ligands are a frequent exception, leaving the electron pair of the M–NO σ-bond at the metal and thus making a backbond of it.

## Details of the applied methods

### Details of the X-ray investigations

The standard X-ray analysis of a small crystal was performed on a Bruker D8venture (APEX3 software, Bruker AXS area detector, Bruker TXS rotating anode). Multiscan absorption correction was applied by using SADABS.[17] The structures were solved by direct methods (SHELXT) and refined by full-matrix least-squares calculations on $F^2$ (SHELX supported by ShelXle).[3b, 18] Thermal ellipsoids were plotted with ORTEP3.[19] CCDC 2190374 contain the supplementary crystallographic data for this paper. These data are provided free of charge by the Cambridge Crystallographic Data Centre (www.ccdc.cam.ac.uk/structures).

The photophysical experiment were done on an Agilent microfocus supernova diffractometer (absorption correction: Gaussian, CrysAlis PRO 1.171.40.67a, Rigaku Oxford Diffraction, 2019).

The specimens were monoclinic red platelets of the formula $C_{36}H_{30}N_2O_2P_2Ru$, $M_r$ = 685.63, $P\ 2_1/n$, Z = 4, $\rho$ = 1.464 g cm$^{-3}$, MoK$\alpha$ radiation, $\mu$ = 0.64 mm$^{-1}$, $T$ = 100 K.

**Table S1:** Overview.

| | D8Venture | Supernova(GS) | Supernova(photo) |
|---|---|---|---|
| $a$/Å | 9.2255(5) | 9.22200(11) | 9.2775(5) |
| $b$/Å | 36.5078(16) | 36.4379(4) | 36.4176(16) |
| $c$/Å | 9.9088(5) | 9.90412(12) | 9.9290(5) |
| $\beta$/° | 111.201(2) | 111.0381(13) | 111.867(6) |
| $V$/Å$^3$ | 3111.4(3) | 3106.24(7) | 3113.3(3) |
| crystal size/mm | 0.10 × 0.08 × 0.01 | 0.68 × 0.50 × 0.15 | 0.29 × 0.15 × 0.12 |
| $T_{max}$, $T_{min}$ | 0.7454, 0.5868 | 1.000, 0.401 | 1.000, 0.535 |
| sin$\theta/\lambda_{max}$/Å$^{-1}$ | 0.627 | 0.650 | 0.759 |
| refls. measured | 28609 | 25704 | 10613 |
| independent refls. | 6271 | 7107 | 10613 |
| reflexes with $I \geq 2\sigma(I)$ | 5430 | 6973 | 8641 |
| $R_{int}$ | 0.035 | 0.0549 | 0.046 |
| parameters | 388 | 391 | 379 |
| $R(F_{obs})$ | 0.073 | 0.041 | 0.047 |
| $R_w(F^2)$ | 0.142 | 0.093 | 0.088 |
| $S$ | 1.33 | 1.01 | 1.10 |
| max. electron density in e Å$^{-3}$ | 1.495 | 0.86 | 0.70 |
| min. electron density in e Å$^{-3}$ | −1.625 | −1.19 | −0.82 |

**Parameters of the photocrystallographic investigation: the GS**

The crystal structure of $[Ru(NO)_2(PPh_3)_2]$ complex consists of distorted tetrahedral conformation (Figure 1 in main text) in which two trans phosphine ligands and two apical nitrosyl groups are centered with ruthenium atom.

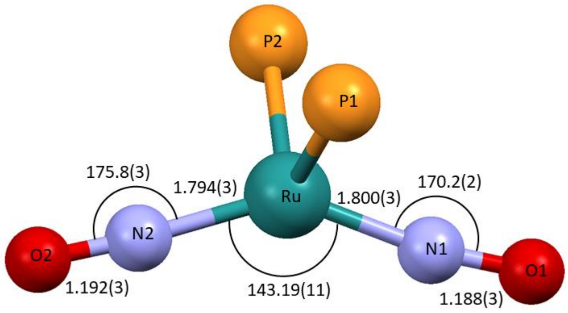


**Figure S2.** The selective representation of atoms of $[Ru(NO)_2(PPh_3)_2]$ complex with their bond distances (Å) and bond angles (°) in the ground state showing distorted tetrahedral geometry.

The nitrosyl N2–O2 is coordinated approx. linearly with the Ru–N–O bond angle 175.8(3)°, while nitrosyl N1–O1 is coordinated with the bond angle of 170.2(2)° showing a small but significant deviation from linearity. Ru–N distances are almost equal with 1.800(3) Å and 1.794(3) Å for Ru–N1 and Ru–N2, respectively. The oppositely arranged phosphorous atoms attached tetrahedrally with ruthenium center at 104.61(3) °. The relatively larger N–Ru–N bond angle 143.19(11)° compared to the smaller P–Ru–P angle, gives rise to a distorted tetrahedral geometry.

The crystal structure of $[Ru(NO)_2(PPh_3)_2]$ is stabilized by weak intermolecular interactions involving the two nitrosyl ligands to form a close packing of the molecules (Figure S3). O1 forms bifurcated van-der-Waals interactions C9–H9···O1$^{i}$ and C33–H33···O1$^{ii}$, with intermolecular distance of 2.431 Å and 2.473 Å, respectively, whilst O2 is involved in C27–H27···O2$^{iii}$ 2.657 Å interactions. Symmetry codes: $^{i}$ $-x, -y + 1, -z + 1$; $^{ii}$ $x - 1, y, z$; $^{iii}$ $x + 1/2, -y + 3/2, z + 1/2$.

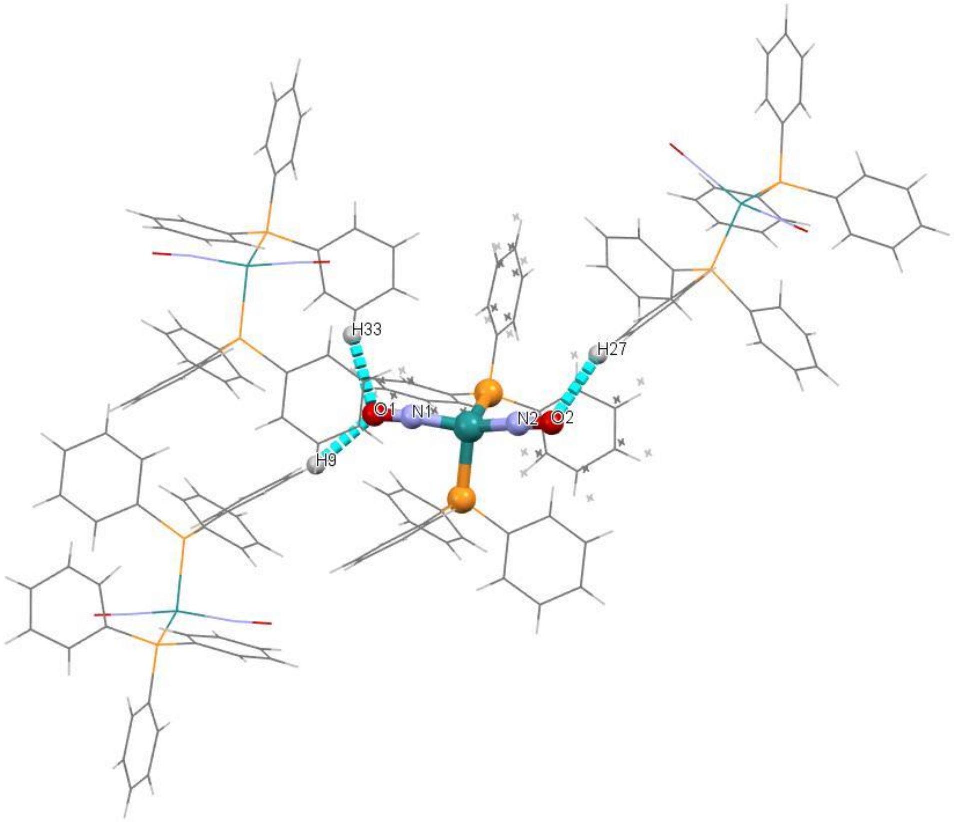


**Figure S3.** Selective intermolecular interactions involving the nitrosyl ligands in the ground state of $[Ru(NO)_2(PPh_3)_2]$.

### Parameters of the photocrystallographic investigation: the PLI state

X-ray diffraction measurements were performed after photoexcitation at 100 K with 590 nm. The photodifference map (Figure 2 in the main text) shows several important features. Strong negative difference electron density is observed at the position of N1–O1 and N2–O2 of the GS, while strong positive peaks appear at nearby positions corresponding to a change of orientation of N1–O1 and N2–O2, corresponding to the PLI state.

A structural model was constructed based on the qualitative information obtained from the photodifference maps considering two molecular entities, the unreacted GS species and the PLI state (keeping in mind that from IR spectroscopy about 50% population of PLI are expected). Both the nitrosyl ligands are considered as make part of one PLI state. This implies that the structural change to be modelled on one molecule involves new positions for both NO ligands, which then necessarily exhibit the same population. In this respect, several refinement strategies corresponding to the four different possible combinations of κ*N*- and κ*O*-bonded nitrosyls were applied to consider the subtracted electron density from the total occupancy of GS in order to obtain the most relevant three-dimensional structural model in PLI state. Table S2 gives agreement factors and main characteristics of the models tested (nomenclature as in Table 1 of main text).

**Table S2:** Refinement details of $[Ru(NO)_2(PPh_3)_2]$ in the ground state (data up to sin$\theta/\lambda$ = 0.997 $Å^{-1}$) and the photo-irradiated state; refinement with Shelxl.

| | Ground state | Photo-irradiated state | | | |
|---|---|---|---|---|---|
| Refinement strategy | ($l$-NO-κ$N$)$_2$ | ($a$-NO-κ$N$)$_2$ | ($a$-NO-κ$N$) ($a$-NO-κ$O$) | ($a$-NO-κ$O$) ($a$-NO-κ$N$) | ($a$-NO-κ$O$)$_2$ |
| N1A–O1A configuration | | nitrosyl | nitrosyl | nitrosyl | nitrosyl |
| N1B–O1B configuration | | nitrosyl | nitrosyl | isonitrosyl | isonitrosyl |
| N2A–O2A configuration | | nitrosyl | nitrosyl | nitrosyl | nitrosyl |
| N2B–O2B configuration | | nitrosyl | isonitrosyl | nitrosyl | isonitrosyl |
| No. of refined parameters | 381 | 379 | 392 | 392 | 392 |
| $R[F^2 > obs*\sigma(F^2)]$ | 0.066 | 0.049 | 0.048 | 0.048 | 0.049 |
| $wR2[F^2 > obs*\sigma(F^2)]$ | 0.132 | 0.088 | 0.088 | 0.086 | 0.088 |
| $S$ | 1.26 | 1.10 | 1.09 | 1.11 | 1.11 |
| $\Delta\rho_{max}$, $\Delta\rho_{min}$ (e $Å^{-3}$) | 1.09, −4.29 | 0.70, −0.82 | 1.04, −0.82 | 0.75, −0.79 | 0.98, −0.79 |
| Refined population of PLI | | | | | |
| N1AO1A N2AO2A (GS) | | 53.6(4)% | 54.6(4)% | 59.3(4)% | 60.4(4)% |
| N1BO1B N2BO2B (PLI) | | 46.4(3)% | 45.4(4)% | 40.7(4)% | 39.6(4)% |
| $U_{eq}$(Ru) | 0.01243(2) | 0.01622(5) | 0.01622(5) | 0.01623(5) | 0.01623(5) |
| $U_{eq}$(P1) | 0.01099(5) | 0.01368(10) | 0.01370(11) | 0.01371(10) | 0.01370(11) |
| $U_{eq}$(P2) | 0.01170(6) | 0.01336(10) | 0.01333(10) | 0.01332(10) | 0.01333(11) |
| $U_{eq}$(N1A) | 0.0181(2) | 0.0206(6) | 0.0207(6) | 0.0262(7) | 0.0262(7) |
| $U_{eq}$(O1A) | 0.0272(3) | 0.0265(8) | 0.0268(8) | 0.0279(9) | 0.0291(10) |
| $U_{eq}$(N1B) | | 0.0206(6) | 0.0207(6) | 0.0279(9) | 0.0291(10) |
| $U_{eq}$(O1B) | | 0.0265(8) | 0.0268(8) | 0.0262(7) | 0.0262(7) |
| $U_{eq}$(N2A) | 0.0188(2) | 0.0201(6)* | 0.0278(6)* | 0.0203(6)* | 0.0272(6)* |
| $U_{eq}$(O2A) | 0.0351(4) | 0.0291(6)* | 0.0223(6)* | 0.0294(6)* | 0.0238(6)* |
| $U_{eq}$(N2B) | | 0.0201(6)* | 0.0223(6)* | 0.0203(6)* | 0.0238(6)* |
| $U_{eq}$(O2B) | | 0.0291(6)* | 0.0278(6)* | 0.0294(6)* | 0.0272(6)* |

We describe hereafter in detail what we consider the model best fitting the data ($a$-NO-κ$N$)$_2$. Two NO conformations were used to refine the appropriate atomic positions, one corresponds to the GS conformation Ru–N1A–O1A (bond angle of 169.4(5)°, $d_{Ru–N1A}$ = 1.885(5) Å, $d_{N1A–O1A}$ = 1.172(5) Å), Ru–N2A–O2A (bond angle of 174.2(5)°, $d_{Ru–N2A}$ = 1.763(5) Å, $d_{N2A–O2A}$ = 1.154(6) Å) with an occupancy factor of 53.6(4)%, and the other corresponds to the excited-state conformation Ru–N1B–O1B (bond angle of 119.1(5)°, $d_{Ru–N1B}$ = 1.732(4) Å, $d_{N1B–O1B}$ = 1.212(6) Å), Ru–N2B–O2B (bond angle of 135.2(5)°, $d_{Ru–N2B}$ = 1.835(5) Å, $d_{N2B–O2B}$ = 1.262(6) Å) with an occupancy factor of 46.4(4)%, respectively. The final refined model is depicted in Figure S4. When the refinement is performed without constraining the occupancies of the two NO groups in the PLI to the same value, one obtains 46.5(3)% for N1BO1B and 59.3(2)% N2BO2B. However, no improvement in agreement values is obtained, and ADP values of involved atoms (N1A, N1B, O1A, N2A, N2B, O2A, O2B) increase by 0.002 to 0.003 $Å^2$ compared to those given in Table S2.

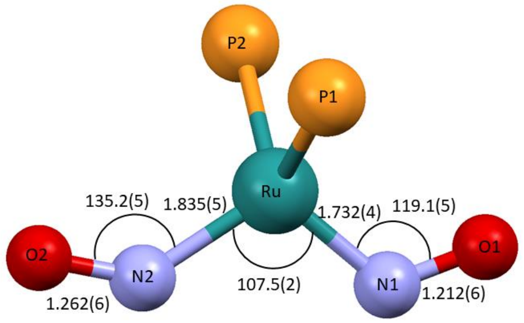


**Figure S4.** Structural model of the PLI state upon photo-excitation at $T$ = 100 K.

The major structural features of the PLI are the bent configuration of both nitrosyl ligands with the bond angles of Ru–N1B–O1B (119.1(5)°) and Ru–N2B–O2B (135.2(5)°), much lower than the ground state of 170.2(2)° and 175.8(3)°, respectively. Almost all the atomic distances and angles of the first Ru coordination are changed by the isomerisation. The refinement of the PLI state as nitrosyl in case of Ru–N1B–O1B and Ru–N2B–O2B results in quite consistent atomic displacement parameters N1BO1B [$N1B_{Ueq}$ = 0.0206(6) Å$^2$, $O1B_{Ueq}$ = 0.0265(8) Å$^2$] and N2BO2B [$N2B_{Uiso}$ = 0.0201(6) Å$^2$, $O2B_{Uiso}$ = 0.0291(6) Å$^2$].

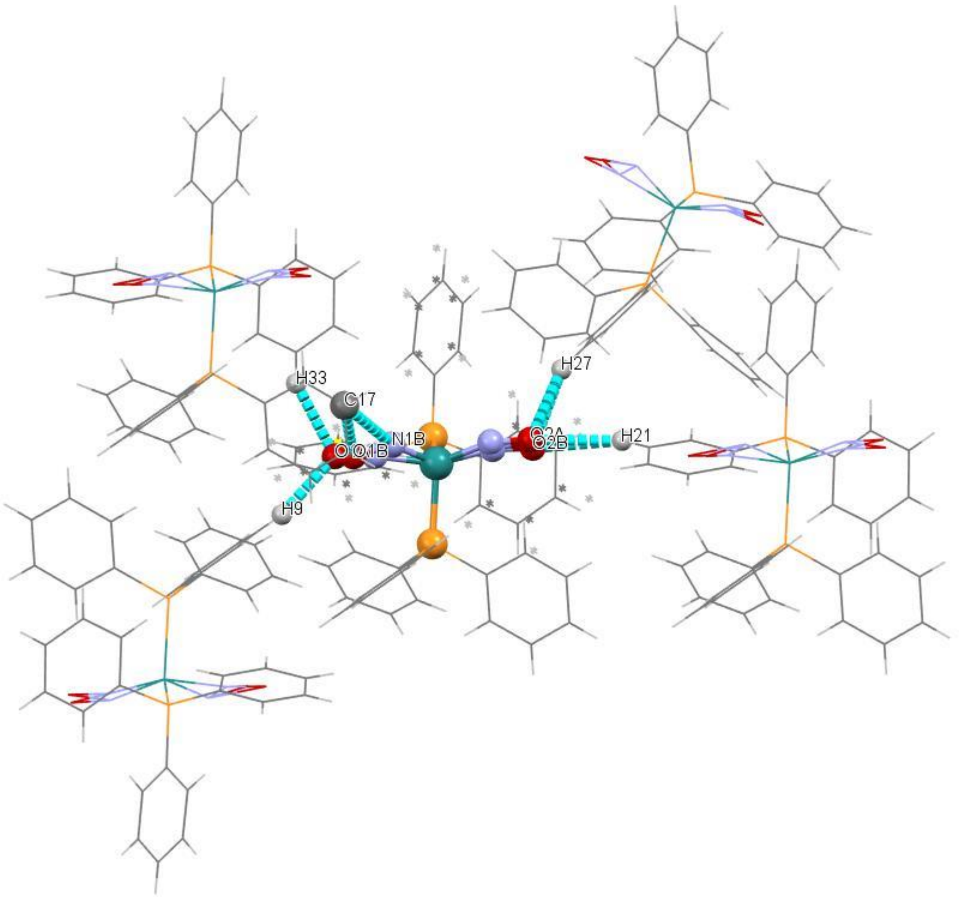


**Figure S5:** Selective intermolecular interactions involving the nitrosyl ligands in the PLI state compared to the GS of $[Ru(NO)_2(PPh_3)_2]$.

The intermolecular interactions change from GS to PLI (Figure S5). The key interactions involved in the PLI state of the photoactive nitrosyl groups are C--H···O, C···N and C···O. In the GS the strongest intermolecular interaction involving the NO group is C9–H9···O1$^{i}$ with an intermolecular distance of 2.431 Å that has been significantly decreased to 2.360 Å after photo-excitation. This suggests that the interactions in the PLI state slightly gets stronger in contrast to the interactions in the GS, which might help stabilize the PLI configuration. Correspondingly, the hydrogen bonding moderately gets stronger in C33– H33···O1$^{ii}$ and C27–H27···O2$^{iii}$ from 2.473 Å and 2.657 Å in the GS to 2.416 Å and 2.652 Å, respectively, in the excited state. The newly introduced moieties O1B, O2B are stabilized by forming the C18--H18···O2B$^{iv}$ ($d_{H18\cdots O2B}$ = 2.570 Å), C27--H27···O2B$^{iii}$ ($d_{H27\cdots O2B}$ = 2.638 Å), C21—H21···O2B$^{v}$ ($d_{H21\cdots O2B}$ = 2.641Å) and C33—H33···O1B$^{ii}$ ($d_{H33\cdots O1B}$ = 2.680 Å). Beside these the C···N and C···O interactions are also formed with distances ranging from 3.161 Å to 3.401 Å and 3.218 Å to 3.4875 Å, respectively.

### Refinement details for the other possible PLI configurations

**1. N1O1 as bent nitrosyl and N2O2 as a bent isonitrosyl: (*a*-NO-κ*N*)(*a*-NO-κ*O*)**

In this model the PLI of N1O1 is a bent nitrosyl while N2O2 is a bent isonitrosyl. The GS configuration for N1O1 was assigned as Ru–N1A–O1A and in PLI the configuration is Ru–N1B–O1B, while the configuration for N2O2 in GS Ru–N2A–O2A and in PLI as Ru–O2B–N2B. The results of the structural refinement lead to a refined population of PLI N1BO1B = 46.0(4)%, N2BO2B = 35.3(15)%, agreement statistic factors of R = 0.048 and wR2 = 0.088 respectively.

When restraining the population of both the PLI to the same value, the structural refinement results in a population of 45.4(4)% for PLI and of 54.6(4)% for GS with the agreement statistics R = 0.048, wR2 = 0.088. The refinement leads further to reasonable values for the atomic displacement parameters (in Å$^2$) N1B = 0.0207(6), O1B = 0.0268(8), N2B = 0.0223(6), O2B = 0.0278(6). However, we note that in this model, the ADP of O2B is larger than that of N2B, even though the O2B is the inner atom of the Ru-O2B-N2B PLI configuration. This is a typical sign of a wrong assignment of the atom species in NO linkage isomers. The oxygen has a higher number of electrons than the nitrogen. So if an oxygen atom is wrongly placed on a nitrogen position, the overstimation (model versus data) of the electron density is somewhat compensated by increasing the ADP value, leading to a smearing of the electron density of the O2B atom. The inverse situation is observed at the outer position, occupied by the N2B, where the model underestimates the experimental electron density and therefore leads to a smaller ADP value. An in-depth discussion of this issue is available.[20]

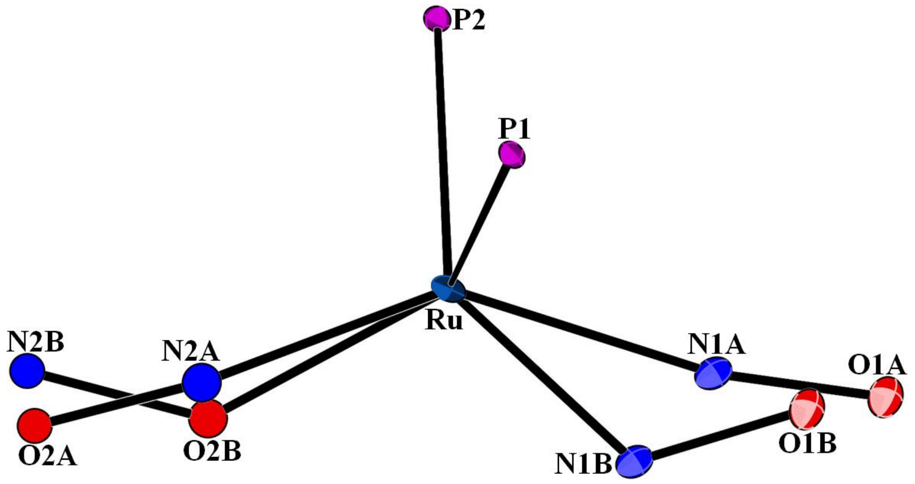


**Figure S6.** Ortep view of N1O1 as bent and N2O2 as isonitrosyl. Ellipsoid sare plotted at 50% probability level.

## 2. N1O1 as isonitrosyl and N2O2 as bent nitrosyl: (*a*-NO-κ*O*)(*a*-NO-κ*N*)

In this model the PLI of N1O1 is a bent isonitrosyl while N2O2 is as bent nitrosyl. The GS configuration for N1O1 was assigned as Ru–N1A–O1A and in PLI the configuration is Ru–O1B–N1B, while the configuration for N2O2 in GS Ru–N2A–O2A and in PLI as Ru–N2B–O2B. The results of the structural refinement lead to a refined population of PLI N1BO1B = 39.9(4)%, N2BO2B = 57.8(18)%, agreement statistic factors of R = 0.048 and wR2 = 0.086, respectively.

When restraining the population of both the PLI to the same value, the structural refinement results in a population of 40.7(4)% for the PLI and of 59.3(4)% for the GS with agreement statistics R = 0.048, wR2 = 0.086. The refinement leads further to reasonable values for the atomic displacement parameters (in $Å^2$) N1B = 0.0279(9), O1B = 0.0262(7), N2B = 0.0203(6), O2B = 0.0294(6). As in the previous model with one isonitrosyl configuration we observe an ADP value for O1B which is too high (compared to GS) and almost identical to the one of the N1B atom, pointing again to a probably wrong assignment N/O of this group.

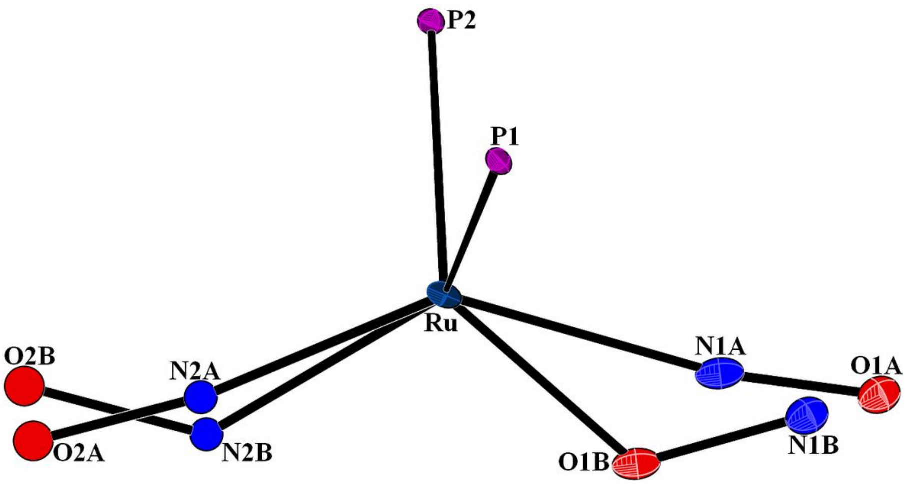


**Figure S7.** Ortep view of N1O1 as bent and N2O2 as isonitrosyl. Ellipsoids are plotted at 50% probability level.

## 3. N1O1 and N2O2 as bent isonitrosyl: (*a*-NO-κ*O*)$_2$

In this model of the PLI the bent isonitrosyl configurations were used for both N1O1 and N2O2. The GS configuration for N1O1 was assigned as Ru–N1A–O1A and in PLI the configuration is Ru–N1B–O1B, while the configuration for N2O2 in GS Ru–N2A–O2A and in PLI as Ru–O2B–N2B. The results of the structural refinement lead to a refined population of PLI N1BO1B = 40.0(4)%, N2BO2B = 35.2(15)%, agreement statistic factors of R = 0.049 and wR2 = 0.088, respectively.

When restraining the population of both the PLI to the same value, the structural refinement results in a population of 39.6(4)% for the PLI and of 60.4(4)% for the GS with agreement statistics R = 0.049, wR2 = 0.088. The refinement leads further to reasonable values for the atomic displacement parameters (in $Å^2$) N1B = 0.0291(10), O1B = 0.0262(7), N2B = 0.0238(6), O2B = 0.0272(6). As in the previous models with one isonitrosyl configuration we observe an ADP value for O2B which is too high and an ADP of N1B which is too low, pointing again to a probably wrong assignment N/O of this group. For the N1BO1B group the effect is less pronounced, but compared to GS the value of the ADP of O1B is still to high.

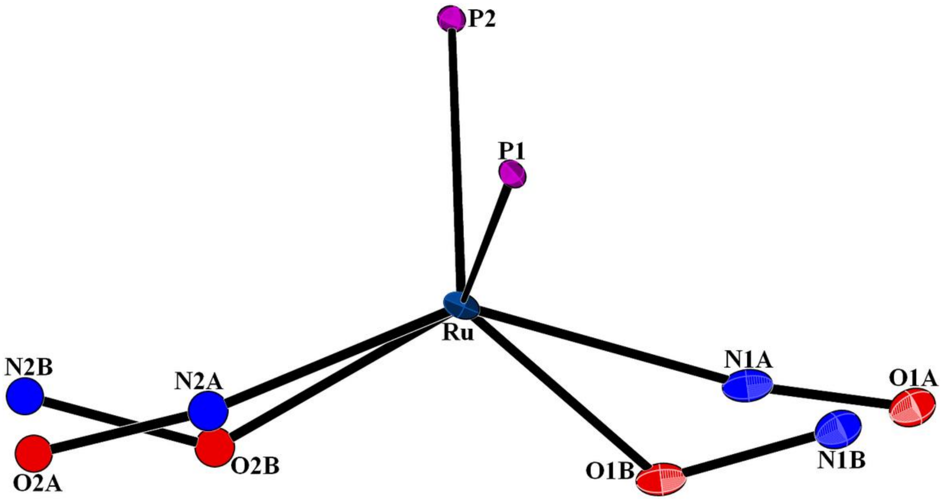


**Figure S8.** Ortep view of N1O1 and N2O2 as isonitrosyl. Ellipsoids are plotted at 50% probability level.

#### Detailed IR spectra

In the GS, the NO ligands have stretching modes associated with the bands at 1612 cm$^{-1}$ and 1657 cm$^{-1}$ at 100 K, in good agreement with the results of Gaughan et al., 1974, which reported the ν(NO) bands at 1615 cm$^{-1}$ and 1665 cm$^{-1}$.[21] In order to find out the optimal spectral range for photo-excitation, a wavelength range of 365–735 nm was tested systematically. The maximal photo-excitation was reached between 556 nm and 590 nm (Figure S9).

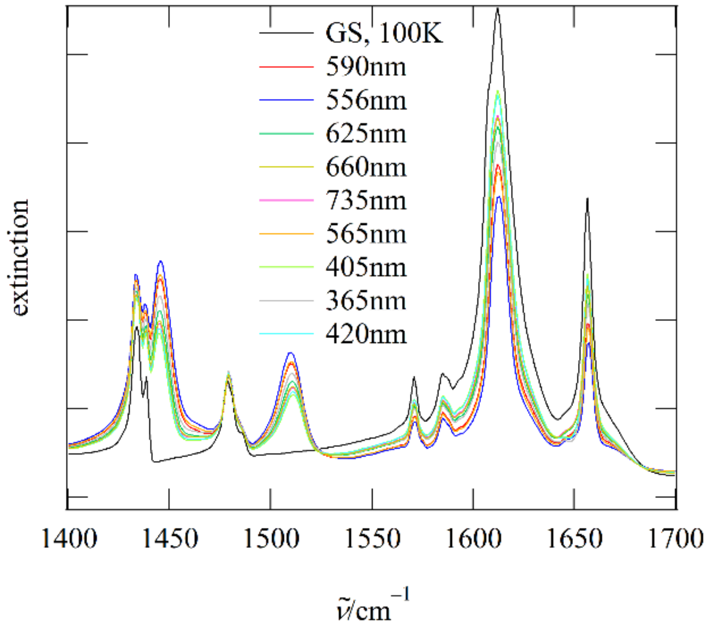


**Figure S9.** IR spectra of $[Ru(NO)_2(PPh_3)_2]$ at 100 K and after irradiation with light of different wavelengths, indicating that the maximum population can be obtained using 556 nm wavelength.

Figure S10 illustrates the effect of light irradiation on the example of 556 nm wavelength. Both GS bands at 1612 cm$^{-1}$ and 1657 cm$^{-1}$ decrease, while two new bands arise at 1445 cm$^{-1}$ and 1510 cm$^{-1}$. Assuming $\nu(NO)_{sym}/\nu(NO)_{asym}$ at 1657/1612 cm$^{-1}$ for GS and at 1510/1445 cm$^{-1}$ for the PLI, we obtain the difference in vibrational frequencies of $\Delta\nu$ = –167 cm$^{-1}$ and $\Delta\nu$ = –147 cm$^{-1}$. From the decrease of

the area of the GS bands at 1612 cm$^{-1}$ and 1657 cm$^{-1}$, the total photoinduced state population is found to be 50%.

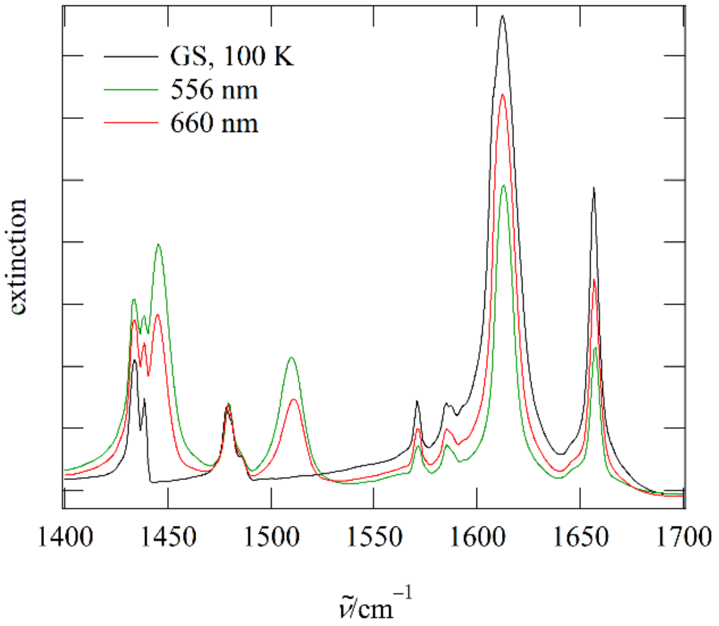


**Figure S10.** IR spectra of $[Ru(NO)_2(PPh_3)_2]$ at 100 K in the ground state (GS, black line) and after irradiation with light of 556 nm wavelength (green line) and after subsequent irradiation with 660 nm (red line).

The PLI state generated by irradiation in the yellow green spectral range can be transferred back (partially) to the GS by irradiation with red light. Figure S10 illustrates this effect for the subsequent irradiation with 556 nm and 660 nm. Both bands of PLI at 1510 cm$^{-1}$ and 1445 cm$^{-1}$ decrease and correspondingly both bands of the GS at 1612 cm$^{-1}$ and 1657 cm$^{-1}$ increase synchronously, indicating a single PLI state.

The radiationless thermally activated decay of the PLI state was examined by collecting IR spectra upon heating the sample after irradiation at low temperature. Both bands of PLI at 1510 cm$^{-1}$ and 1445 cm$^{-1}$ decrease and correspondingly both bands of the GS at 1612 cm$^{-1}$ and 1657 cm$^{-1}$ increase synchronously. The relaxation is completed at 220 K (see Figure S11).

The population behavior was monitored as a function of irradiation fluence $Q = I\,t$ for the irradiation wavelength 556 nm. Figure S12 shows the corresponding spectra, where a synchronous decrease of the two GS bands is observed together with the increase of the two PLI bands. Analysis of the area $A$ of these four bands as a function of $Q$ shows a mono-exponential behavior $A = A_0\left(1 - exp(-Q/Q_0)\right)$ with the same time constant $Q_0$ = 1.2(1) J cm$^{-2}$ for all bands (Figure S13).

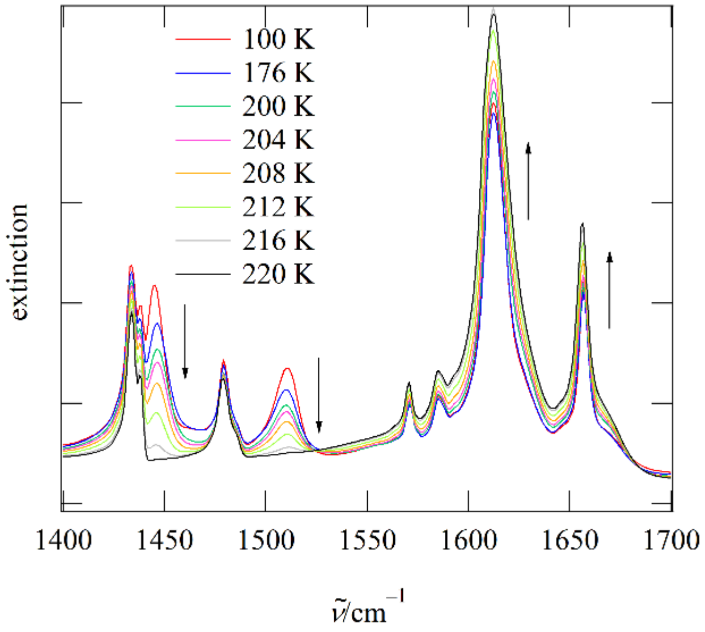


**Figure S11:** IR spectra of $[Ru(NO)_2(PPh_3)_2]$ at 100 K and after irradiation with light of 556 nm wavelength and at different temperature upon heating indicating the decrease of the PLI bands around 210–220K and the transfer back to GS.

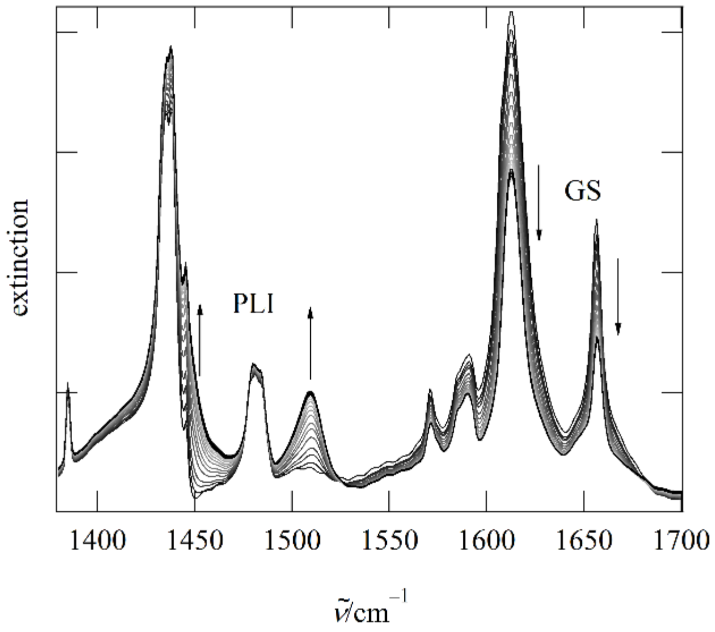


**Figure S12:** IR spectra of $[Ru(NO)_2(PPh_3)_2]$ at 100 K as a function of irradiation time using 556 nm wavelength illustrating increase of two PLI bands at 1510 $cm^{-1}$ and 1445 $cm^{-1}$ and decrease of GS bands at 1612 $cm^{-1}$ and 1657 $cm^{-1}$.

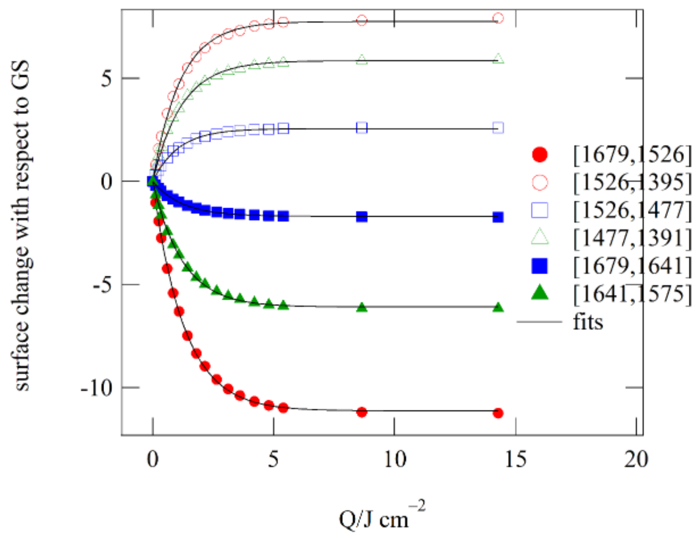


**Figure S13:** Band areas of PLI and GS bands integrated over spectral ranges as indicated in brackets, as a function of fluence $Q = I\ t$. The solid line corresponds to a mono-exponential fit.

### Details of the calorimetric investigation

DSC measurements allow for the determination of the activation energy and thus determination of the energy barrier separating the PLI from the GS.[2] Figure S14 shows the result of the corresponding measurement and analysis supposing an Arrhenius like behavior, yielding an activation energy of 0.63(1) eV. Note that we observe only one decay indicative of a single PLI state.

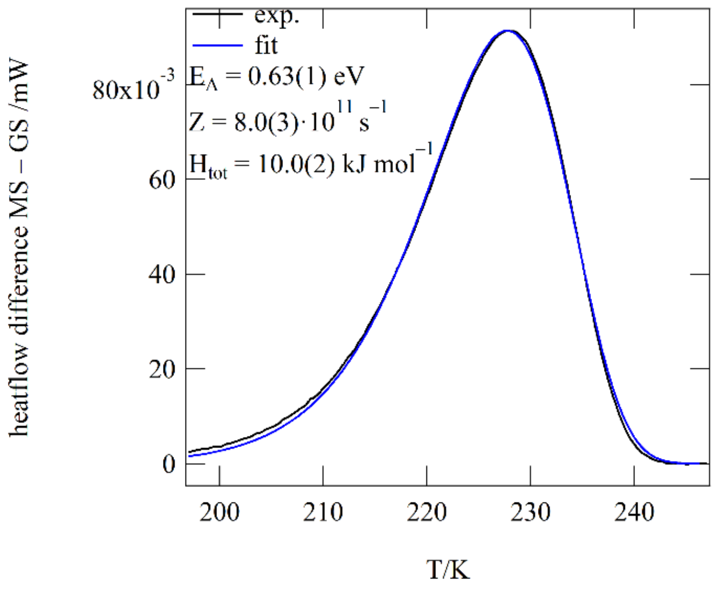


**Figure S14**: Difference of heat flow (black line) measured before and after light irradiation. Fit assuming an Arrhenius like decay, yielding an activation energy of 0.63(1) eV.

**Details of the UV/Vis spectroscopic investigation**

UV/Vis spectroscopy yields insight into the electronic structure of PLI, especially concerning the optimal wavelength range for the population and depopulation of the linkage isomers. Figure S15 shows the result of the corresponding measurement on KBr pellets of **1**. We observe that the GS bands (at 540 nm and 450 nm) decrease and new bands of MS arise (clearly visible at 485 nm, probably also a weak one above 600 nm). This is consistent with the observation of the maximum population by irradiation between 556-590 nm (see Fig. S9).

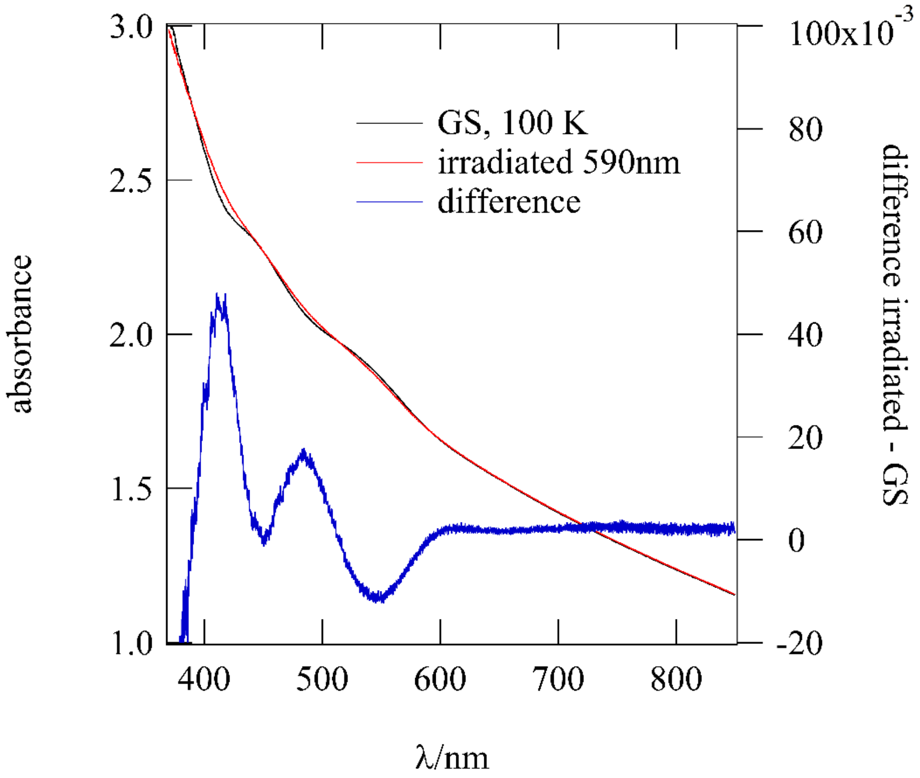


**Figure S15**: UV/Vis spectra in the range 850-370 nm, before and after irradiation with 590 nm with the corresponding difference spectrum, indicating the decrease of GS bands and increase of MS bands.

**Details of the computational analysis**

**Table S3 is an extended version of Table 1 of the main text:**

**Table S3:** Isomer energies, relative to *E*(GS) = 0 with GS = (*l*-NO-κ*N*)$_2$, and the calculated energies of the N–O stretches on the BP86/def2-TZVP+D3 level of theory (analytical frequencies with Orca5). More or less decoupling of the symmetric/asymmetric N–O stretches is covered by footnotes. For an extended version of the table, see the SI.

| | *E*/eV | *E*/kJ mol$^{-1}$ | $\tilde{\nu}_{sym}$/cm$^{-1}$ | $\tilde{\nu}_{asym}$/cm$^{-1}$ |
|---|---|---|---|---|
| experiment: | | | | |
| (*l*-NO-κ*N*)$_2$ | 0 | 0 | 1657 | 1612 |
| (*a*-NO-κ*N*)$_2$ | – | – | 1510 | 1445 |
| **(NO-κ*N*)$_2$ isomers:** | | | | |
| (*l*-NO-κ*N*)$_2$ | 0 | 0 | 1709 | 1676 |
| (*a*-NO-κ*N*)$_2$ | 0.631 | 60.9 | 1553 | 1519 |
| (*s*-NO-κ*N*)$_2$ | 1.180 | 113.8 | 1508 | 1463 |
| (*a*-NO-κ*N*)(*s*-NO-κ*N*) | → (*l*-NO-κ*N*)$_2$ | | | |
| **(NO-κ*N*)(NO-κ*O*) isomers:** | | | | |
| (*l*-NO-κ*N*)(*l*-NO-κ*O*) | 1.975 | 190.6 | 1672[a] | 1619[b] |
| (*a*-NO-κ*N*)(*a*-NO-κ*O*) | → (*l*-NO-κ*N*)(NO-κ$^2$*N*,$\underline{O}$) | | | |
| (*s*-NO-κ*N*)(*s*-NO-κ*O*) | → (*a*-NO-κ*N*)$_2$ | | | |
| (*a*-NO-κ*N*)(*s*-NO-κ*O*) | → (*l*-NO-κ*N*)(*l*-NO-κ*O*) | | | |
| (*s*-NO-κ*N*)(*a*-NO-κ*O*) | → (*l*-NO-κ*N*)(*l*-NO-κ*O*) | | | |
| **(NO-κ*O*)$_2$ isomers:** | | | | |
| (*l*-NO-κ*O*)$_2$ | 4.122 | 397.7 | 1607 | 1591 |
| (*a*-NO-κ*O*)$_2$ | 4.355 | 420.2 | 1575 | 1547 |
| (*s*-NO-κ*O*)$_2$ | → (*l*-NO-κ*O*)(NO-κ$^2$$\underline{N}$,*O*) | | | |
| (*a*-NO-κ*O*)(*s*-NO-κ*O*) | → (*l*-NO-κ*O*)$_2$ | | | |
| **(*l*-NO)(NO-κ$^2$*N*,*O*) isomers:** | | | | |
| (*l*-NO-κ*N*)(NO-κ$^2$$\underline{N}$,*O*) | 1.135 | 109.5 | 1661[c] | 1282[d] |
| (*l*-NO-κ*N*)(NO-κ$^2$*N*,$\underline{O}$) | 2.205 | 212.7 | 1677[c] | 1395[d] |
| (*l*-NO-κ*O*)(NO-κ$^2$$\underline{N}$,*O*) | 3.133 | 302.3 | 1631[e] | 1265[d] |

[a] largely NO-κ*N*, [b] largely NO-κ*O*, [c] *l*-NO-κ*N*, [d] NO-κ$^2$*N*,*O*, [e] *l*-NO-κ*O*.

**Table S4:** Experimental vs. calculated data (*method*/def2-TZVP[+D3], $r^2$SCAN-3c with mTZVPP); mean values for geometrical data. The '+ tol' column refers to the BP86/def2-TZVP+D3+CPCM(toluene) level; the 'exp.' column refers to Fig. S2.

| | exp. | BP86 | + tol | $BP86_{zora}$ | $TPSS_{zora}$ | $r^2$SCAN-3c |
|---|---|---|---|---|---|---|
| ($l$-NO-κ$N$)$_2$: | | | | | | |
| $d_{N–O}$/Å | 1.190 | 1.190 | 1.193 | 1.192 | 1.189 | 1.179 |
| $d_{Ru–N}$/Å | 1.797 | 1.797 | 1.795 | 1.770 | 1.771 | 1.799 |
| $\tilde{\nu}_{sym}$/cm$^{-1}$ | 1657 | 1709 | 1678 | 1717 | 1723 | 1769 |
| $\tilde{\nu}_{asym}$/cm$^{-1}$ | 1612 | 1676 | 1632 | 1687 | 1689 | 1735 |

**Table S5:** Transition energies, relative to $E$(GS) = 0. TS is the transition state between the global ground-state ($l$-NO-κ$N$)$_2$ and the ($a$-NO-κ$N$)$_2$ PLI isomer. The computational level is BP86/ZORA-def2-TZVP//*method*/ZORA-def2-TZVP (D3 applied to both the structural optimisation and the respective single-point energy). $E_a$ is $E$(TS) − $E$(MS).

| | TS | | MS | | $E_a$ | |
|---|---|---|---|---|---|---|
| $E$ in: | eV | kJ mol$^{-1}$ | eV | kJ mol$^{-1}$ | eV | kJ mol$^{-1}$ |
| exp. (DSC) | | | | | 0.63(1) | 61(1) |
| BP86 | 1.240 | 119.6 | 0.611 | 59.0 | 0.628 | 60.6 |
| TPSS | 1.284 | 123.9 | 0.692 | 66.8 | 0.592 | 57.1 |
| TPSSh | 1.322 | 127.6 | 0.745 | 71.9 | 0.577 | 55.7 |
| ωB97M-V | 1.333 | 128.6 | 0.843 | 81.3 | 0.490 | 47.3 |
| W6B95 | 1.317 | 127.1 | 0.753 | 72.7 | 0.564 | 54.4 |
| PWPB95 | 1.543 | 148.9 | 0.762 | 73.5 | 0.781 | 75.4 |

**Table S6:** Extension to Table 4 in the main text. Instead of mean values, individual numbers are given for NO(1) (subscript 1) and NO(2) (subscript 2).

| | GS | TS | MS |
|---|---|---|---|
| $Q_1/e$ | –0.532 | –0.402 | –0.473 |
| $Q_2/e$ | –0.556 | –0.400 | –0.499 |
| $\lambda_1$(in-plane-$\pi^*_{NO\ 1}$) | 0.442 | 0.483 | 0.410 |
| $\lambda_2$(in-plane-$\pi^*_{NO\ 2}$) | 0.444 | 0.479 | 0.413 |
| $\lambda_1$(out-of-plane-$\pi^*_{NO\ 1}$) | 0.415 | 0.263 | 0.377 |
| $\lambda_2$(out-of-plane-$\pi^*_{NO\ 2}$) | 0.424 | 0.265 | 0.385 |
| $\lambda_1$(3$\sigma_{NO\ 1}$) | 0.855 | 0.899 | 0.908 |
| $\lambda_2$(3$\sigma_{NO\ 2}$) | 0.857 | 0.901 | 0.911 |
| $\tilde{\nu}^a{}_1$(N–O)/cm$^{-1}$ | 1572 | 1546 | 1452 |
| $\tilde{\nu}^a{}_2$(N–O)/cm$^{-1}$ | 1561 | 1539 | 1435 |
| $k^a{}_1$(Ru–N)/N cm$^{-1}$ | 4.338 | 2.256 | 2.811 |
| $k^a{}_2$(Ru–N)/N cm$^{-1}$ | 4.398 | 2.496 | 2.652 |
| $k^a{}_1$(N–O)/N cm$^{-1}$ | 10.87 | 10.52 | 9.28 |
| $k^a{}_2$(N–O)/N cm$^{-1}$ | 10.73 | 10.42 | 9.06 |

**Table S7:** QTAIM charges for **1**. For comparison with the ZORA-corrected values of the main text, these values are computed on the BP86/def2-TZVP+D3+CPCM(toluene) level by Multiwfn 3.8.[9]

| *Q*/*e* | GS | TS | MS |
|---|---|---|---|
| Ru | 0.616 | 0.467 | 0.592 |
| mean NO | −0.555 | −0.424 | −0.500 |
| mean $PPh_3$ | 0.248 | 0.177 | 0.203 |

**Table S8:** xyz files of the GS, TS and MS, computed on the BP86/def2-TZVP+D3+CPCM(toluene) level of theory.

GS:

```
Ru   0.00757897363283  -0.04928251342466  -0.03084137086808
N   -0.39096216601750  -0.28620042574779  -1.76391380631893
O   -0.55771011451203  -0.34234212146621  -2.94333668419881
N   -0.34387023836494  -0.75571263484110   1.58108158048347
O   -0.47926256754857  -1.23861900614621   2.66553908334573
P    2.30775383926804  -0.17466354727430   0.00740964509475
P   -0.33011600251343   2.23938952393416   0.04495640422532
C    4.81409274103633  -1.09925170810514  -3.14042224796585
C    4.34723418521285  -0.25442931705500  -4.15208023585510
C    4.19837378382062  -1.10073092625548  -1.88598431276324
C    3.25691448203483   0.58603364642063  -3.90717061865843
C    3.10578414988602  -0.25543923582396  -1.63672216825891
C    3.79187306699537  -4.08599179555764   2.00267616754433
C    2.66150143919093  -4.08424534909641   1.17777308832074
C    2.63568370258572   0.58248437541235  -2.65860906029753
C    4.47593647610131  -2.89247670542139   2.24766856307075
C    2.21576507904426  -2.89369317253385   0.60461850268503
C    4.03816421671095  -1.69974962279213   1.66488087108588
C    4.35571674234393   1.77738796671906   0.29057585465823
C    2.90544460624329  -1.69357195721924   0.83851620017171
C    4.97323234431530   2.83903325095948   0.95779029912449
C    3.24231813319990   1.14457051365411   0.86014999233255
C    2.74348468026926   4.13668865108177  -1.92513331536627
C    4.48707724723378   3.27038535990524   2.19545406239204
C    2.26570984974087   4.19866319259206  -3.23726331748001
C    1.94330096708581   3.61669690233646  -0.90747630689190
C    2.77278852133859   1.56623165785391   2.11436991667795
C    3.39162539700212   2.62318874693198   2.77964005051549
C    0.97771471643080   3.73805433919996  -3.52752193510545
C    0.64952060346118   3.15149620393786  -1.19178237193803
C    0.17272612501730   3.21643544585650  -2.51372915780936
C    0.35197761060262   4.44539386696951   1.71297864682187
C   -0.07527335286424   3.11131996201050   1.63514391206270
C    0.49599669164942   5.06723088553025   2.95487885412633
C   -0.38534710398923   2.42212633379007   2.81719954745809
C   -2.05167033321289   2.74039082662522  -0.36684032808918
C    0.19982629675366   4.37010988879163   4.12930721852353
C   -3.03965804589523   1.76536410152311  -0.54118613920481
C   -0.25168134828007   3.04843573659449   4.05631537023008
C   -2.38943586173532   4.09747836508278  -0.49316837812729
C   -4.35348765090808   2.13867252357988  -0.84187544650725
C   -3.69829223408353   4.46910413994299  -0.79763641341589
C   -4.68338232584005   3.48920873576939  -0.97239668562220
H    5.66206711684106  -1.76043714435476  -3.32562441032624
H    4.82854324278651  -0.25655656563488  -5.13115746414095
H    4.56901817977377  -1.75909623361497  -1.09998551233124
H    2.88058525678116   1.24422355176216  -4.69118195942463
H    4.13364025979364  -5.01577213633348   2.45914332189466
H    2.11979507966441  -5.01190115781483   0.98839751602948
H    4.72202015196032   1.45873411851703  -0.68448907023586
H    5.35547683356784  -2.88701120061035   2.89342122182218
```

| | | | |
|---|---|---|---|
| H | 5.82930345426905 | 3.33847488488075 | 0.50200856176984 |
| H | 1.32005022357478 | -2.88221268283487 | -0.01957821475047 |
| H | 4.57926745500448 | -0.77256997998640 | 1.85305918202147 |
| H | 1.77963441125777 | 1.22908658854017 | -2.47771997885800 |
| H | 3.74905511670920 | 4.48606943138773 | -1.68875758817009 |
| H | 2.89677284180844 | 4.59811770489564 | -4.03189670171760 |
| H | 4.95993485959158 | 4.11072001881570 | 2.70549668500288 |
| H | 2.33307998878661 | 3.55941819336265 | 0.10709381657791 |
| H | 1.90405586490111 | 1.07819823672543 | 2.55417297691592 |
| H | 3.00535630139506 | 2.95367504230616 | 3.74377276492769 |
| H | 0.59810069133076 | 3.77791349952320 | -4.54940249311153 |
| H | 0.58648380951429 | 5.00132494897311 | 0.80602493101853 |
| H | -0.82448530919007 | 2.84502376021907 | -2.74962814199257 |
| H | 0.84288036009023 | 6.10026035823600 | 3.00242129358208 |
| H | -2.76793665384619 | 0.71192938066284 | -0.44328059089469 |
| H | -0.72449141923621 | 1.38831798217927 | 2.76361393341752 |
| H | 0.31854105338124 | 4.85540622121403 | 5.09912897327711 |
| H | -0.49146356566024 | 2.49850696160127 | 4.96708711574436 |
| H | -1.62604356552517 | 4.86532166740677 | -0.35900296132064 |
| H | -5.11658904434863 | 1.37112989190411 | -0.97870002869592 |
| H | -3.95254029626092 | 5.52530483243291 | -0.90025057833075 |
| H | -5.70648902115932 | 3.78239472139284 | -1.21199612990894 |

TS:

| | | | |
|---|---|---|---|
| Ru | -0.04909016913768 | -0.01710614813438 | 0.08082418610850 |
| N | 1.21785012000749 | 1.19624964555304 | -0.56109722816125 |
| O | 1.35005217138207 | 2.12443317829051 | -1.29402551816691 |
| N | -0.12071178451136 | -0.00918420960246 | 1.94824067978838 |
| O | -0.90375331969839 | 0.11481044416236 | 2.83642758191547 |
| P | -1.98902546159523 | 0.93186113343846 | -0.45400032212072 |
| P | -0.28209525011483 | -2.05778709313593 | -1.04310769001880 |
| C | -2.91937475713209 | 3.73623909560499 | -3.35983488826380 |
| C | -2.19169055873900 | 3.27160534062938 | -4.46047195460937 |
| C | -2.85946998019642 | 3.05528381870189 | -2.14119380688610 |
| C | -1.39397591968981 | 2.13030361401601 | -4.33506499047746 |
| C | -2.06466202434552 | 1.90523440980921 | -2.01222076027066 |
| C | -3.12719071327036 | 4.13313607124762 | 2.71711694342522 |
| C | -1.85535417995681 | 4.11185028952043 | 2.13118806730028 |
| C | -1.32328620203078 | 1.45847782252604 | -3.11407781189945 |
| C | -4.08000049752332 | 3.18509677077236 | 2.33832104257473 |
| C | -1.54083833947570 | 3.14324644241972 | 1.17944699050410 |
| C | -3.76718991547243 | 2.21826923603381 | 1.37657078291643 |
| C | -4.46213210507090 | 0.04601989174748 | -1.57910441179740 |
| C | -2.49538199571542 | 2.18864478552475 | 0.79048060391554 |
| C | -5.53949546541503 | -0.84024225878340 | -1.66280033000936 |
| C | -3.49698192442203 | -0.11004669530732 | -0.57396878307695 |
| C | -2.76024595182727 | -1.68333480752483 | -4.30552146998317 |
| C | -5.66627595598087 | -1.88335945531202 | -0.74042055737084 |
| C | -1.85920889919563 | -1.50123613617791 | -5.35808172473134 |
| C | -2.29385798737011 | -1.92402028431118 | -3.01328332714930 |
| C | -3.64710683139793 | -1.14235217356629 | 0.36486915177888 |
| C | -4.72353548902998 | -2.02500535870581 | 0.28374262900559 |
| C | -0.48465836311881 | -1.56985079176026 | -5.11175059900182 |
| C | -0.91470327112880 | -1.98606192299869 | -2.75609183720015 |
| C | -0.01326786493461 | -1.81092728600340 | -3.81975516926876 |
| C | -2.06033092566664 | -4.28573958969324 | -0.89069757170363 |
| C | -1.29170357474730 | -3.33642495137757 | -0.20319434109626 |
| C | -2.80578251517264 | -5.23266932309002 | -0.18402896946740 |
| C | -1.25986321731667 | -3.36562929340834 | 1.19952274195083 |
| C | 1.32044589412168 | -2.94007364756300 | -1.27033064292846 |
| C | -2.77959384865594 | -5.24913022343291 | 1.21286603173710 |
| C | 2.52249660209778 | -2.26564880456263 | -1.02278524680876 |
| C | -1.99632263775500 | -4.31787622143080 | 1.90371155559054 |
| C | 1.35775528447190 | -4.27531280842453 | -1.70455325183567 |

| | | | |
|---|---|---|---|
| C | 3.74795056924746 | -2.91239624477579 | -1.21136262475302 |
| C | 2.58018199978059 | -4.92065816966596 | -1.89094788712045 |
| C | 3.77867038845322 | -4.23947459956500 | -1.64494933872084 |
| H | -3.53397984225116 | 4.63336627567887 | -3.44914398065001 |
| H | -2.24041983307750 | 3.80395818571335 | -5.41176175851976 |
| H | -3.43096824212844 | 3.41872561691436 | -1.28649646904928 |
| H | -0.81941387306060 | 1.76327471803218 | -5.18658985431478 |
| H | -3.37046963104619 | 4.88469597686624 | 3.46996327964483 |
| H | -1.10454020888095 | 4.84758160874797 | 2.42413320170192 |
| H | -4.35521600450349 | 0.84188397173762 | -2.31551670688165 |
| H | -5.07225466815697 | 3.19393300065314 | 2.79215978998238 |
| H | -6.27589460694295 | -0.72177387642357 | -2.45977301606938 |
| H | -0.54369991278315 | 3.11660103940187 | 0.73730748332162 |
| H | -4.51811815208249 | 1.48444904096461 | 1.08356155729819 |
| H | -0.69176602761366 | 0.57685145603820 | -3.01046371103678 |
| H | -3.83527099034373 | -1.62898157219088 | -4.48456132769831 |
| H | -2.22637960148622 | -1.30411747022521 | -6.36602091906768 |
| H | -6.49919191169940 | -2.58403339751724 | -0.81858717624644 |
| H | -3.00633848394008 | -2.05364414412763 | -2.20197636847777 |
| H | -2.89929412620232 | -1.26685662816813 | 1.14734386513681 |
| H | -4.81345422187182 | -2.83545113745460 | 1.00838311447890 |
| H | 0.22622533721901 | -1.43240126268251 | -5.92789146816400 |
| H | -2.09011895516408 | -4.27988187841246 | -1.98017627149324 |
| H | 1.06108295600801 | -1.85264704239187 | -3.63383902197603 |
| H | -3.41034271272990 | -5.95869598807519 | -0.72985576549961 |
| H | 2.49314825694368 | -1.22806651928569 | -0.68310144121636 |
| H | -0.66268735683488 | -2.62721364603274 | 1.73783042357973 |
| H | -3.36754444735087 | -5.98524633271507 | 1.76312310035033 |
| H | -1.96631529153694 | -4.32610779249016 | 2.99464464214958 |
| H | 0.42914098124673 | -4.81500641803319 | -1.89568064732296 |
| H | 4.67796588466989 | -2.37688687071615 | -1.01548772894955 |
| H | 2.59910238939231 | -5.95811737447158 | -2.22736673880966 |
| H | 4.73399114745873 | -4.74629742301842 | -1.78886501081356 |

MS:

| | | | |
|---|---|---|---|
| Ru | -0.00628840123138 | 0.08300873980587 | -0.18139640838058 |
| N | 1.29041579548019 | 0.96110568992822 | -1.12559173950124 |
| O | 1.37842545455586 | 1.12413627182298 | -2.31714497501530 |
| N | 0.50208211872381 | 0.20049479735471 | 1.57190682409345 |
| O | -0.11560816607723 | -0.19381076053189 | 2.53563869019153 |
| P | -2.10169069962314 | 1.05177356447103 | -0.50815177162957 |
| P | -0.20784910596936 | -2.07578872753240 | -1.03478203687170 |
| C | -2.78403883749651 | 4.06724978648660 | -3.24654179464188 |
| C | -2.28236288714813 | 3.50874963509563 | -4.42636851693136 |
| C | -2.73625074084469 | 3.34504615728135 | -2.05149071955523 |
| C | -1.72190682838194 | 2.22775377908305 | -4.40692683634915 |
| C | -2.18026293784697 | 2.05700015428956 | -2.03091280975936 |
| C | -3.32118517001292 | 4.03725411773985 | 2.81976869861268 |
| C | -1.97840237632704 | 3.87571995828368 | 2.46267858068205 |
| C | -1.66189450661389 | 1.50756839143732 | -3.21413161421350 |
| C | -4.30844752092596 | 3.28294938392241 | 2.17924047328313 |
| C | -1.62351194925209 | 2.95797737068474 | 1.47319096982132 |
| C | -3.95666283572739 | 2.37209751252661 | 1.18062973124936 |
| C | -4.47139021259394 | 0.02408923130115 | -1.69736415184552 |
| C | -2.61076340474458 | 2.20581010737505 | 0.82037273547424 |
| C | -5.49982052888903 | -0.91696285679169 | -1.79921495372933 |
| C | -3.54670379963623 | -0.05904163839375 | -0.64845300462880 |
| C | -2.74854596177333 | -1.81399488571786 | -4.25913833676567 |
| C | -5.61021116003636 | -1.94301224665381 | -0.85662741940954 |
| C | -1.87130566489264 | -1.53396070367228 | -5.31077744970149 |
| C | -2.25641120794053 | -2.04862842703330 | -2.97527015479284 |
| C | -3.67810242426014 | -1.07582915932699 | 0.31083950004243 |
| C | -4.70297692667893 | -2.01428509920009 | 0.20747619687869 |
| C | -0.49386845617275 | -1.49215881126434 | -5.07330378266185 |

| | | | |
|---|---|---|---|
| C | -0.87355091740208 | -2.01171424490655 | -2.72982489496554 |
| C | 0.00532716999526 | -1.72889528294685 | -3.79115343814696 |
| C | -1.94247997137770 | -4.32214442753781 | -0.74964963243174 |
| C | -1.22838375348047 | -3.28968516546348 | -0.12469425712872 |
| C | -2.68282237869937 | -5.22524596794506 | 0.01533159891007 |
| C | -1.24458294991029 | -3.18919264600833 | 1.27516234494773 |
| C | 1.37137660703615 | -2.98957707050580 | -1.25169621271728 |
| C | -2.70633438187499 | -5.11397780523369 | 1.40893079522261 |
| C | 2.55684883068248 | -2.45315146204405 | -0.73714404799497 |
| C | -1.97976571306693 | -4.09793201554392 | 2.03771141057903 |
| C | 1.40132233465173 | -4.22552244916398 | -1.91826944249971 |
| C | 3.76415767705794 | -3.14290874522329 | -0.88762965549209 |
| C | 2.60580472491363 | -4.91131258908882 | -2.06980546642100 |
| C | 3.78991746699130 | -4.36992922265558 | -1.55397624562741 |
| H | -3.21573846925585 | 5.06883850483923 | -3.25444454692491 |
| H | -2.32058051604578 | 4.07479302158423 | -5.35836887220937 |
| H | -3.13215255426652 | 3.78245802737504 | -1.13508880113802 |
| H | -1.32048808128950 | 1.78695202653034 | -5.32028479786252 |
| H | -3.59686922515458 | 4.74452717392951 | 3.60374679600602 |
| H | -1.20356128616059 | 4.45589251210694 | 2.96526389106963 |
| H | -4.37312227937404 | 0.80951783094760 | -2.44639855616227 |
| H | -5.35646784556393 | 3.40123254276820 | 2.45877527688362 |
| H | -6.21006480963459 | -0.85407538042589 | -2.62547477465183 |
| H | -0.57485537798730 | 2.80904089686461 | 1.20806437786266 |
| H | -4.72923015656587 | 1.78670265537674 | 0.68129988238396 |
| H | -1.20976809761828 | 0.51745280350338 | -3.20258493559837 |
| H | -3.82524568441876 | -1.84041640592622 | -4.43287338825179 |
| H | -2.26026053313070 | -1.34205821335622 | -6.31146478442406 |
| H | -6.40368490394622 | -2.68644734309538 | -0.94836032291402 |
| H | -2.95041850224579 | -2.24723887236094 | -2.16023319183608 |
| H | -2.96131756195184 | -1.14155232521703 | 1.13020383325416 |
| H | -4.77962355215687 | -2.81297604790852 | 0.94643158359778 |
| H | 0.19691762392024 | -1.26814631136554 | -5.88726006404063 |
| H | -1.93366200227497 | -4.41781213646244 | -1.83541138018865 |
| H | 1.07958689606095 | -1.68098368208533 | -3.60923813560641 |
| H | -3.24393835261379 | -6.01854336736208 | -0.48089317174148 |
| H | 2.52662364019061 | -1.48948308468133 | -0.22499043479687 |
| H | -0.69597148373158 | -2.38532208939334 | 1.76882946582735 |
| H | -3.28912155978998 | -5.81896869726114 | 2.00367431059456 |
| H | -1.98992959406347 | -4.00644613616553 | 3.12516674552714 |
| H | 0.48210668339187 | -4.64958077515648 | -2.32480345552356 |
| H | 4.68454298357820 | -2.71704558383470 | -0.48620626857738 |
| H | 2.62291413916090 | -5.87002188504801 | -2.58975723452436 |
| H | 4.73195705975906 | -4.90702089722353 | -1.67368282621292 |

## References


[1] Ruthenium Complexes, Chapter 8 in *Inorg. Synth.* (Ed.: T. B. Rauchfuss), Wiley, **2010**, 148-163.

[2] D. Schaniel, T. Woike, L. Tsankov, M. Imlau, *Thermochim. Acta* **2005**, *429*, 19-23.

[3] a) G. Sheldrick, *Acta Crystallogr., Sect. A,* **2008**, *64*, 112-122; b) G. Sheldrick, *Acta Crystallogr., Sect. C,* **2015**, *71*, 3-8.

[4] a) F. Neese, Wiley Interdisciplinary Reviews: Computational Molecular Science **2018**, 8, e1327; b) F. Neese, Wiley Interdisciplinary Reviews: Computational Molecular Science **2012**, 2, 73-78.

[5] F. Weigend, R. Ahlrichs, *PCCP* **2005**, *7*, 3297-3305.

[6] F. Weigend, *PCCP* **2006**, *8*, 1057-1065.

[7] a) S. Grimme, J. Antony, S. Ehrlich, H. Krieg, *J. Chem. Phys.* **2010**, *132*, 154104; b) S. Grimme, S. Ehrlich, L. Goerigk, *J. Comput. Chem.* **2011**, *32*, 1456-1465.

[8] F. Neese, F. Wennmohs, A. Hansen, U. Becker, *Chem. Phys.* **2009**, *356*, 98-109.

[9] T. Lu, F. Chen, *J. Comput. Chem.* **2012**, *33*, 580-592.

[10] P. Salvador, E. Ramos-Cordoba, M. Gimferrer, M. Montilla, *Universitat de Girona, Spain* **2010**.

[11] J. Popp, T. Riggenmann, D. Schröder, T. Ampßler, P. Salvador, P. Klüfers, *Inorg. Chem.* **2021**, *60*, 15980-15996.

[12] H. J. B. Marroux, B. F. E. Curchod, C. A. Faradji, T. A. Shuttleworth, H. A. Sparkes, P. G. Pringle, A. J. Orr-Ewing, *Angew. Chem. Int. Ed.* **2017**, *56*, 13713-13716.

[13] P. Karen, *Angew. Chem. Int. Ed.* **2015**, *54*, 4716-4726.

[14] T. Ampßler, G. Monsch, J. Popp, T. Riggenmann, P. Salvador, D. Schröder, P. Klüfers, *Angew. Chem. Int. Ed.* **2020**, *59*, 12381-12386.

[15] a) M. Gimferrer, J. Van der Mynsbrugge, A. T. Bell, P. Salvador, M. Head-Gordon, *Inorg. Chem.* **2020**, *59*, 15410-15420; b) V. Postils, C. Delgado-Alonso, J. M. Luis, P. Salvador, *Angew. Chem. Int. Ed.* **2018**, *57*, 10525-10529; c) E. Ramos-Cordoba, V. Postils, P. Salvador, *J. Chem. Theory Comput.* **2015**, *11*, 1501-1508.

[16] J. H. Enemark, R. D. Feltham, *Coord. Chem. Rev.* **1974**, *13*, 339-406.

[17] L. Krause, R. Herbst-Irmer, G. M. Sheldrick, D. Stalke, *J. Appl. Crystallogr.* **2015**, *48*, 3-10.

[18] a) G. M. Sheldrick, *Acta Crystallogr., Sect. A*, **2015**, *71*, 3-8; b) C. B. Hübschle, G. M. Sheldrick, B. Dittrich, *J. Appl. Crystallogr.* **2011**, *44*, 1281-1284.

[19] L. Farrugia, *J. Appl. Crystallogr.* **2012**, *45*, 849-854.

[20] B. Cormary, I. Malfant, M. Buron-Le Cointe, L. Toupet, B. Delley, D. Schaniel, N. Mockus, T. Woike, K. Fejfarová, V. Petříček, M. Dušek, *Acta Crystallographica Section B* **2009**, *65*, 612-623.

[21] A. P. Gaughan, B. J. Corden, R. Eisenberg, J. A. Ibers, *Inorg. Chem.* **1974**, *13*, 786-791.
