# Supplementary figures and images for: Pas de Deux of an NO Couple: Synchronous Photoswitching from a Double‐Linear to a Double‐Bent Ru(NO)2 Core under Nitrosyl Charge Conservation
Source: Angew Chem Int Ed Engl. 2022 Sep 15;61(42):e202210671. doi: 10.1002/anie.202210671 (PMC9826364; doi:10.1002/anie.202210671)

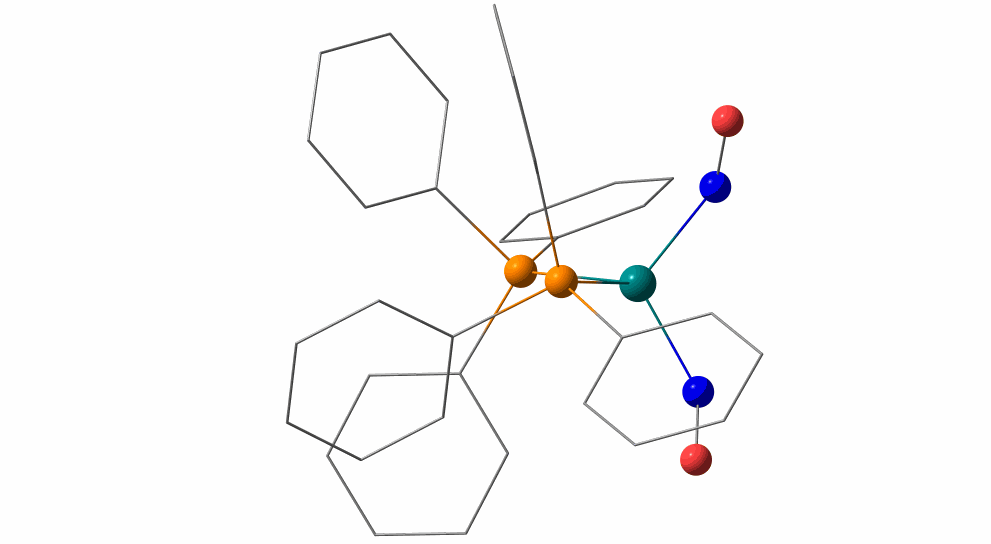

Supplement: Supplementary file 2 — Supporting Information [file ANIE-61-0-s001.gif]
